# Risk factors for musculoskeletal injuries in the military: a qualitative systematic review of the literature from the past two decades and a new prioritizing injury model

**DOI:** 10.1186/s40779-021-00357-w

**Published:** 2021-12-10

**Authors:** Stefan Sammito, Vedran Hadzic, Thomas Karakolis, Karen R. Kelly, Susan P. Proctor, Ainars Stepens, Graham White, Wes O. Zimmermann

**Affiliations:** 1 Section Experimental Aerospace Medicine Research, German Air Force Centre of Aerospace Medicine, Flughafenstraße 1, 51147 Cologne, Germany; 2 Occupational Medicine, Faculty of Medicine, Otto-Von-Guericke-University of Magdeburg, 39120 Magdeburg, Germany; 3 Faculty of Sport, University of Ljubljana, 1000 Ljubljana, Slovenia; 4 Defence Research and Development Canada, Toronto, ON M3K 2C9 Canada; 5 Warfighter Performance, Naval Health Research Center, San Diego, CA 92106-3599 USA; 6 Military Performance Division, US Army Research Institute of Environmental Medicine, Natick, MA 01760 USA; 7 Research Service, VA Boston Healthcare System, Boston, MA 02130 USA; 8 Centre for Military Medicine Research, Riga Stradins University, Riga, 1007 Latvia; 9 Human and Social Sciences Group, Defense Science and Technology Laboratory, Portsdown Hill Road, Fareham, PO17 6AD UK; 10 Department of Military Sports Medicine, Royal Netherlands Army, 3584 AB Utrecht, The Netherlands; 11 Department of Military/Emergency Medicine, Uniformed Services University of the Health Sciences, Bethesda, MD 20814 USA

**Keywords:** Military, Musculoskeletal injuries, Risk factors, Prevention, Intervention, Injury

## Abstract

**Background:**

Musculoskeletal injuries (MSkIs) are a leading cause of health care utilization, as well as limited duty and disability in the US military and other armed forces. MSkIs affect members of the military during initial training, operational training, and deployment and have a direct negative impact on overall troop readiness. Currently, a systematic overview of all risk factors for MSkIs in the military is not available.

**Methods:**

A systematic literature search was carried out using the PubMed, Ovid/Medline, and Web of Science databases from January 1, 2000 to September 10, 2019. Additionally, a reference list scan was performed (using the “snowball method”). Thereafter, an international, multidisciplinary expert panel scored the level of evidence per risk factor, and a classification of modifiable/non-modifiable was made.

**Results:**

In total, 176 original papers and 3 meta-analyses were included in the review. A list of 57 reported potential risk factors was formed. For 21 risk factors, the level of evidence was considered moderate or strong. Based on this literature review and an in-depth analysis, the expert panel developed a model to display the most relevant risk factors identified, introducing the idea of the “order of importance” and including concepts that are modifiable/non-modifiable, as well as extrinsic/intrinsic risk factors.

**Conclusions:**

This is the qualitative systematic review of studies on risk factors for MSkIs in the military that has attempted to be all-inclusive. A total of 57 different potential risk factors were identified, and a new, prioritizing injury model was developed. This model may help us to understand risk factors that can be addressed, and in which order they should be prioritized when planning intervention strategies within military groups.

**Supplementary Information:**

The online version contains supplementary material available at 10.1186/s40779-021-00357-w.

## Background

Musculoskeletal injuries (MSkIs) are a leading cause of health care utilization, as well as limited duty and disability in the US military [1] and other armed forces [2–6]. MSkIs affect members of the military during initial training [7], operational training [8], and deployment [9], and have a direct negative impact on overall troop readiness. MSkIs have been shown to make up 50% of disease and non-battle injury (DNBI) casualties, and 43% of DNBI casualties requiring evacuation. Additionally, many service members sustain MSkIs, which are treated conservatively in the theater during deployment, but eventually require surgery following a combat tour [10, 11]. The consequences of MSkIs are reduced individual fitness and health [12], and ultimately discharge from military duty [13, 14].

As such, the prevention of MSkIs is considered a main target area to increase the readiness, performance, and health of military personnel. Approaches include the identification of risk factors and purposeful intervention strategies to reduce MSkIs. In recent decades, hundreds of original studies have been published with the goal of identifying risk factors for MSkIs, including narrative and systematic reviews on specific risk factors [15–26]. However, an overall summary of the published data on risk factors for MSkIs in the military is not available. Further, for several risk factors, such as sex, there is an ongoing debate on whether there is a direct association with an increased risk of MSkIs, or whether the association is indirect via a confounding risk factor [27]. Finally, there is no model that clarifies the relative order of importance of the risk factors for MSkIs in the military.

Given the gaps in knowledge identified above and the fact that soldier readiness is of great importance to all allied militaries, the multidisciplinary NATO Science and Technology Organization (STO) Research Task Group (RTG) 283 on “Reducing musculoskeletal injuries” set out to perform a systematic review of risk factors for MSkIs in the military to address and discuss the facilitation of successful interventions.

## Methods

A systematic literature search considering the PRISMA guidelines [28] was initiated using the PubMed, Ovid/Medline, and Web of Science databases with the search terms “(military) AND ((injury) OR (trauma)) AND ((basic training) OR (physical training))” with all MeSH terms (see details on Additional file 1) on September 10, 2019. The principal criterion for inclusion was that the study reported on risk factors for MSkIs in a military population. The exclusion criteria were as follows: a language other than English; studies without a risk factor evaluation; and studies published before January 1, 2000. Review articles (without a meta-analysis) were used to find the included original works (see below), but were not included as such in this review. Of the 1794 studies identified (after removing duplicates), 179 were selected for full-text analysis. After full-text analysis, 42 papers were excluded because they did not meet the inclusion criteria, and 19 studies were reviews and did not present new information. So far, a total of 118 original papers and 3 meta-analyses have been included.

**Fig. 1:**
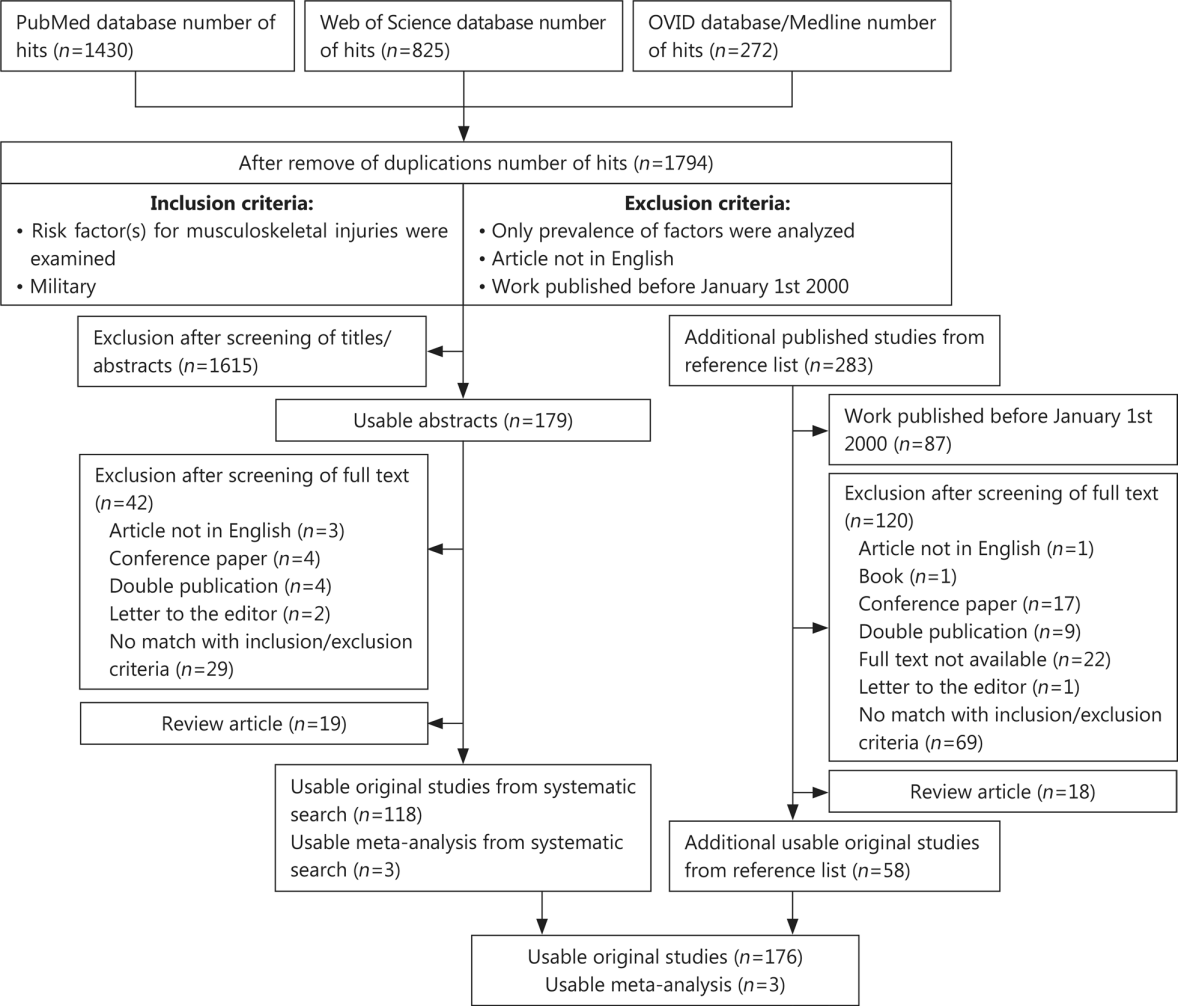
Flowchart of the systematic review

Moreover, to present a complete overview, a reference list scan (using the “snowball method”) [29] was performed on each of the 179 fully analyzed texts, including each of the 19 review articles. With this approach, an additional 283 studies were identified, of which 87 were excluded due to the publication date being before January 1, 2000. The remaining 196 papers were also read in full to determine relevance. If two studies reported on exactly the same population, only the publication that provided the most details was included. As a result, an additional 58 studies were included in this review, bringing the total to 176 original papers and 3 meta-analyses (Fig. 1).

Once all the literature was identified, a list of all reported risk factors was created. Each original paper and meta-analysis was then assigned to a risk factor. If an original paper described multiple risk factors, it was assigned to every risk factor it reported.

In the results section, a general description of all the included publications is provided first, followed by specific descriptions per risk factor. Risk factors were sorted into different groups (in alphabetical order): lifestyle factors, medical factors, occupational factors, physiological factors, social factors, and training factors. For each risk factor, an accompanying table was included that summarizes each aspect of the supporting studies: lead author; year of publication; country of origin; characteristics of the population examined (branch and unit/type of military activity); study type (retrospective or prospective); sample size of the population studied; and whether or not the study concluded that the risk factor was correlated to MSkIs (yes or no). In a number of publications, more than one risk factor was evaluated.

Finally, the multidisciplinary expert panel (consisting of all coauthors of this review) classified the evidence supporting the association between a risk factor and MSkI into one of five categories: strong, moderate, weak, insufficient, or no evidence. For this classification, the expert panel took into account the results of the studies, as well as the number of participants and their professional experience in military MSkI injury prevention. In addition, the expert panel included a determination as to whether a risk factor would be considered modifiable or non-modifiable in the military context. A risk factor was defined as modifiable if a service member could influence it (e.g., to be a smoker) or if military authorities could influence it (e.g., by changing the training schedule or by providing other gear). Risk factors classified as non-modifiable are beyond personal control (e.g., the weather). Whether a risk factor is modifiable is a significant determinant for the application of intervention strategies. Based on the literature review and an in-depth analysis, the multidisciplinary expert panel developed a model to classify the different risk factors identified, introducing the concept of “order of importance” and including the notions of modifiable/non-modifiable and extrinsic/intrinsic risk factors.

## Results

Of the 176 original papers, 101 came from investigations in the US Armed Forces. Additional investigations were conducted in the armed forces of the UK (19 studies), Israel (18 studies), and Finland (14 studies). Australia and Switzerland produced 4 studies each, China and Greece had 3 studies each, Germany had 2 studies, and Belgium, Denmark, India, Iran, Malta, Poland, Slovenia, and Sweden were represented by 1 study each. A majority of the studies examined risk factors in the army (113 studies), whereas there were considerably fewer studies conducted in the marines (16 studies), the air force (7 studies), the navy (5 studies), and the special operations forces (2 studies). Seven studies explored risk factors, including multiple armed services branches; 4 studies were conducted only among recruits or participants in academy training, and 22 studies did not include descriptions of the particular service branch. More than half of the studies (*n* = 101) chose a prospective study design, and the remaining 75 papers evaluated data retrospectively. The study populations ranged from 20 subjects [30] to 5,580,875 analyzed person-years [31]. In two studies [32, 33], no information about the underlying size of the population was reported. Less than half of the studies (*n* = 79) scrutinized populations of less than 1000 participants, while 27 studies had a population greater than 10,000 participants. A number of retrospective studies involved populations with over 100,000 participants [31, 34–51]. A large minority of the studies included both male and female military personnel (*n* = 51). In 33 studies, only male members were included, whereas 17 studies focused exclusively on women in the military. In most of the studies (*n* = 75), no specific information was given about the sex of the included participants.

### Lifestyle factors

#### Alcohol intake

Nine studies focused on higher alcohol intake as a risk factor for MSkIs (Table 1). Five studies were conducted in the US Army, 2 within the British Army, and 1 in Finland and in Greece. The sizes of the study populations ranged from 64 to 4139 participants. Three of the 9 studies identified alcohol intake as a risk factor for MSkIs, and 6 did not show a significant association between alcohol intake and MSkIs.

**Table 1:** Summary of studies that focused on alcohol intake, calcium intake, milk consumption, vegetable consumption, vegetarian diet, sleep time, and smoking as risk factors for MskIs

Study	Publication year	Country	Branches	Unit/training	Study type	*n*	Risk factor^*^
Alcohol intake							
Canham-Chervak [52]	2006	USA	Army	Recruits	P	1156 M, 746 F	No
Chatzipapas [53]	2008	Greece	n/a	Active duty	R	64	No
Cosio-Lima [54]	2013	USA	Army	Sergeants Major Academy	R	149	No
Lappe [55]	2005	USA	Army	Recruits BCT	R	4139 F	Yes (F)
Lappe [56]	2001	USA	Army	Recruits BCT	P	3758 F	Yes (F)
Robinson [57]	2016	UK	Army	Recruits	P	1810	No
Schneider [58]	2000	USA	Army	Airborne Div	R	1214	Yes
Taanila [59]	2012	Finland	Army	Conscripts	P	982 M	No (M)
Wilkinson [60]	2009	UK	Army	Infantry	P	660	No
Calcium intake (low)							
Chatzipapas [53]	2008	Greece	n/a	Active duty	R	64	No
Givon [61]	2000	Israel	n/a		P	2306 M	No (M)
Moran [62]	2012	Israel	Army	Recruits of elite combat unit	P	116	No
Moran [63]	2012	Israel	Army	Elite combat unit BCT	P	74	Yes
Milk consumption (low)							
Cosman [64]	2013	USA	Army	Military Academy	P	755 M, 136 F	No
Moran [62]	2012	Israel	Army	Recruits of elite combat unit	P	116	No
Sanchez-Santos [65	2017	UK	Marines	Recruits	P	1082 M	Yes (M)
Vegetables consumption							
Robinson [57]	2016	UK	Army	Recruits	P	1810	No
Sanchez-Santos [65]	2017	UK	Marines	Recruits	P	1082 M	No (M)
Vegetarian diet							
Dash [66]	2012	India	Army	Recruits	P	8570	Yes
Sleep time (reduced)							
Kovcan [67]	2019	Slovenia	Army	Infantry, active duty	R	118 M, 11 F	No
Wyss [68]	2014	Switzerland	Army	Recruits BCT	P	1676	Yes
Smoking							
Altarac [69]	2000	USA	Army	Recruits	P	187 M, 915 F	Yes
Anderson [70]	2015	USA	Army	Light Infantry Brigade	R	2101	Yes
Anderson [71]	2017	USA	Army	Light Infantry	R	4384 M, 363 F	No
Bedno [72]	2013	USA	Army	IET	P	8456 M	Yes
Bedno [35]	2019	USA	Army	Recruits BCT	R	238,772	Yes
Brooks [73]	2019	USA	Army	Recruits BCT	R	1460 M, 540 F	Yes
Canham-Chervak [52]	2006	USA	Army	Recruits	P	1156 M, 746 F	Yes
Chatzipapas [53]	2008	Greece	n/a	Active duty	R	64	No
Cosio-Lima [54]	2013	USA	Army	Sergeants Major Academy	R	149	No
Cosman [64]	2013	USA	Army	Military Academy	P	755 M, 136 F	Yes
Cowan [74]	2012	USA	Army	Trainees	P	1568 F	No
Cowan [75]	2011	USA	Army	Recruits	P	7323	Yes
Davey [76]	2015	UK	Marines		P	1090 M	Yes
Fallowfield [77]	2018	UK	Air Force	Recruits	P	990 M, 203 F	Yes
Givon [61]	2000	Israel	n/a		P	2306 M	Yes (less)
Grier [78]	2017	USA	Army	Infantry brigades	R	4236 M	No
Grier [79]	2010	USA	Multiple		R	24,177 M	Yes
Kelly [80]	2000	USA	Navy	Recruits BCT	R	86 F	No
Knapik [81]	2010	USA	Air Force	Recruits BCT	P	1042 M, 375 F	Yes
Knapik [82]	2013	USA	Army	Army military police training	P	1838 M, 553 F	Yes^##^
Knapik [83]	2013	USA	Army	Brigade Combat Team^#^	P	805	No
Knapik [84]	2007	USA	Army	Band	R	159 M, 46 F	No
Knapik [85]	2001	USA	Army	Recruits	P	182 M, 168 F	Yes
Knapik [86]	2008	USA	Army	Paratrooper training	R	1677	No
Knapik [87]	2008	USA	Army	Recruits BCT	P	2147 M, 920 F	Yes
Knapik [88]	2009	USA	Marines	Recruits BCT	P	840 M, 571 F	Yes (M), No (F)
Korvala [89]	2010	Finland	n/a	Conscripts	P	192	No
Lappe [55]	2005	USA	Army	Recruits BCT	R	4139 F	Yes
Kovcan [67]	2019	Slovenia	Army	Infantry, active duty	R	118 M, 11 F	Yes
Lappe [56]	2001	USA	Army	Recruits BCT	P	3758 F	Yes
Lauder [90]	2000	USA	Army	Active duty	P	230 F	No (F)
Munnoch [91]	2007	UK	Marines		P	1115 M	Yes
Nagai [92]	2017	USA	Army	Airborne Div	P	275	Yes
Pihlajamäki [93]	2019	Finland	n/a		R	4029 M	No
Psaila [94]	2017	Malta	n/a	Recruits BCT	P	114 M, 13 F	No
Rappole [95]	2017	USA	Army	Army Brigade	R	1099	Yes
Reynolds [96]	2009	USA	Army	Infantry	P	181	Yes
Reynolds [97]	2002	USA	Army	Construction engineers & Combat artillery soldiers	P	313	No
Reynolds [98]	2000	USA	Marines	Winter mountain training	P	356	Yes
Robinson [57]	2016	UK	Army	Recruits	P	1810	No
Roos [99]	2015	Switzerland	Army	Recruits	P	651 M	Yes
Ruohola [100]	2006	Finland	n/a	Recruits	P	756 M	No
Sanchez-Santos [65]	2017	UK	Marines	Recruits	P	1082 M	No
Scheinowitz [101]	2017	Israel	Army	Recruits	P	350 F	No
Schneider [58]	2000	USA	Army	Airborne Div	R	1214	No
Sharma [102]	2019	UK	Army	Infantry recruits	P	562 M	Yes
Sharma [103]	2011	UK	Army	Infantry recruits	P	468 M	Yes
Taanila [59]	2012	Finland	Army	Conscripts	P	982 M	No (M)
Taanila [104]	2015	Finland	Army	Conscripts	P	1411 M	Yes
Trone [105]	2014	USA	Marine Corp Air Force Army	Recruits BCT	R	900 M, 597 F	Yes
Wang [106]	2003	China	n/a	Military Police Forces Training	R	805 M	No
Wilkinson [60]	2009	UK	Army	Infantry	P	660	No
Wunderlin [107]	2015	Switzerland	Army	Recruits	P	230 M	Yes
Zhao [108]	2016	China	Army	Recruits	P	1398 M	No

*BCT* basis combat training; *n/a* Not available; *R* retrospective study; *P* prospective study; *M* male; *F* female; *(M)* risk factor only for males; *(F)* risk factor only for females

*Risk factor for musculoskeletal injuries (MSkI); ^#^Deployment; ^##^Former smoking

There is insufficient scientific evidence for alcohol intake as a modifiable risk factor.

#### Calcium intake (low)

Four studies focused on low (daily) calcium intake as a risk factor for MSkIs (Table 1). Three studies were conducted in the Israel Defense Force (IDF) and one in the Armed Forces of Greece. The sizes of the study populations ranged from 64 to 2306 participants. Only the study with one of the smallest populations identified low daily calcium intake as a risk factor for MSkIs. The other three studies, including one with more than 2000 participants, did not find a significant association.

There is insufficient scientific evidence for low (daily) calcium intake as a modifiable risk factor.

#### Milk consumption (low)

Three studies focused on milk consumption as a risk factor for MSkIs (Table 1). The research was conducted within the militaries of Israel, the USA, and the UK (1 study from each country). The sizes of the study populations ranged from 116 to 1082 participants. Only one study identified low milk consumption as a risk factor for MSkIs; the other two studies did not find a significant association.

There is insufficient scientific evidence for low milk consumption as a modifiable risk factor.

#### Vegetable consumption

Two studies focused on the amount of vegetables eaten (as measured via a self-report questionnaire) as a risk factor for MSkIs (Table 1). The research was conducted within different branches of the UK military. The sizes of the study populations ranged from 1082 to 1810 participants. Neither study found a significant association between the amount of vegetable consumption and MSkIs.

There is no scientific evidence for the amount of vegetable consumption as a modifiable risk factor for MSkIs.

#### Vegetarian diet

Only one study focused on a vegetarian diet as a risk factor for MSkIs (Table 1). This study was conducted within the Indian Army. In this study, with 8570 participants, a vegetarian diet was identified as a risk factor for stress fractures.

There is weak scientific evidence for a vegetarian diet as a modifiable risk factor.

#### (Reduced) sleep time

Two studies focused on little time for sleep as a risk factor for MSkIs (Table 1). These studies were conducted within the Army of Switzerland and the Army of Slovenia. The sizes of the study populations ranged from 129 to 1676 participants. A larger study identified little time for sleep as a risk factor for MSkIs; however, this was not observed within the smaller study.

There is weak scientific evidence for little time for sleep as a modifiable risk factor.

#### Smoking

Fifty-four studies focused on smoking as a risk factor for MSkIs (Table 1). Most of the research was conducted within different branches of the US Armed Forces (32 studies); additional studies were conducted within the militaries of the UK (8 studies), Finland (5 studies), China, Israel, Switzerland (2 studies from each) and Greece, Malta and Slovenia (1 study from each nation). The study populations ranged from 64 to 238,772 participants. Twenty-seven studies identified smoking as a risk factor for MSkIs, and 23 studies did not find a significant association between smoking and MSkI. One study found a significant increase in MSkIs related to a lower level of smoking, and one study found that former smoking habits were a significant risk factor for MSkIs. In one study, the association between smoking and increased risk for MSkIs was found only for males (not for females). A meta-analysis, which included 18 studies, found that smoking increases the risk for MSkIs, for males by 26% (a low level of smoking) up to 84% (a high level of smoking) and for females by 30% (low level of smoking) up to 56% (high level of smoking) [24]. For both sexes together, the increased risk ranges from 27 to 71%.

There is strong scientific evidence for smoking as a modifiable risk factor for MSkIs. Smoking is associated with a 27–71% increased risk of MSkIs.

### Medical factors

#### Current illness

The term “current illness” was used to describe the situation where an injured person was ill (e.g., with influenza at the time the MSkI occurred). There was only one study on current illness as a risk factor for MSkIs (Table 2). The study was conducted in 2010 in the US Armed Forces. With 24,177 male participants, this study found a significant association between current illness and an increased risk for MSkIs. It must be noted that the risk factor “current illness” may represent a bias. Soldiers with an identified current illness are generally removed from active duty and training. This means that current illness is a risk factor mostly based on retrospective self-report by the service member.

**Table 2:** Summary of studies that focused on current illness, prior pregnancy, prescription of contraceptives, prescription of NSAIDs, previous MSkIs, serum iron/serum ferritin, and vitamin D status as risk factors for MSkIs

Study	Publication year	Country	Branches	Unit/training	Study type	*n*	Risk factor^*^
Current illness							
Grier [79]	2010	USA	Multiple		R	24,177 M	Yes (M)
Prescription of contraceptives							
Knapik [87]	2008	USA	Army	Recruits BCT	P	920 F	No
Knapik [88]	2009	USA	Marines	Recruits BCT	P	571 F	No
Scheinowitz [101]	2017	Israel	Army	Recruits	P	350 F	No
Shaffer [109]	2006	USA	Marines	Recruits BCT	R	2962 F	No
Prescription of NSAID							
Hughes [50]	2019	USA	Army	Active duty	R	120,730	Yes
Previous MSkI							
Cameron [110]	2013	USA	Army	Military Academy	P	630 M, 84 F	Yes
Cosman [64]	2013	USA	Army	Military Academy	P	755 M, 136 F	No
Evans [111]	2005	USA	Army		R	1532	Yes
Finestone [112]	2011	Israel	Army	Elite infantry soldier	P	77 M	No (M)
Garnock [113]	2018	Australia	Navy	Recruits	P	95 M, 39 F	Yes
George [114]	2012	USA	Army	Combat medics	P	1230	Yes
Givon [61]	2000	Israel	n/a		P	2306 M	Yes (M) (invers)
Hill [115]	2013	USA	Army	Active duty	R	83,323	Yes
Knapik [81]	2010	USA	Air Force	Recruits BCT	P	1042 M, 375 F	No
Knapik [82]	2013	USA	Army	Army military police training	P	1838 M, 553 F	Yes (M), No (F)
Knapik [116]	2013	USA	Army	Combat engineer enlisted trainees	P	1633	Yes
Knapik [83]	2013	USA	Army	Brigade Combat Team^#^	P	805	No
Knapik [86]	2008	USA	Army	Paratrooper training	R	1677	Yes
Knapik [87]	2008	USA	Army	Recruits BCT	P	2147 M, 920 F	No (M), Yes (F)
Kovcan [67]	2019	Slovenia	Army	Infantry, active duty	R	118 M, 11 F	Yes
Kucera [117]	2016	USA	Army	Cadets	P	9811	Yes
Lappe [56]	2001	USA	Army	Recruits BCT	P	3758 F	No (F)
Lisman [118]	2013	USA	Marines	Officer candidate training	P	874	Yes
Monnier [119]	2019	Sweden	Marines	Training course	P	48 M, 5 F	Yes
Rice [120]	2017	UK	Marines	Recruits	P	147 M	Yes (M) (invers)
Robinson [57]	2016	UK	Army	Recruits	P	1810	Yes
Roos [99]	2015	Switzerland	Army	Recruits	P	651 M	Yes (M)
Roy [121]	2014	USA	Army	Active duty	R	625 F	Yes (F)
Schneider [58]	2000	USA	Army	Airborne Div	R	1214	Yes
Scott [122]	2015	USA	Army	Reserve Officer Training	R	165 M, 30 F	No
Shaffer [109]	2006	USA	Marines	Recruits BCT	R	2962 F	No (F)
Taanila [123]	2010	Finland	n/a	Conscripts	P	944 M	Yes (M)
Wang [106]	2003	China	n/a	Military Police Forces Training	R	805 M	Yes (M)
Wilkinson [60]	2009	UK	Army	Infantry	P	660	Yes
Zhao [108]	2016	China	Army	Recruits	P	1398 M	Yes^##^ (M)
Prior pregnancy							
Knapik [87]	2008	USA	Army	Recruits BCT	P	920 F	Yes
Serum iron/serum ferritin							
Merkel [124]	2008	Israel	Army	Infantry/non-combatant (medics)	P	83 M, 355 F	Yes
Moran [125]	2008	Israel	Army	Recruits	P	227 F	Yes (F)
Vitamin D status							
Burgi [126]	2011	USA	Navy	Recruits	P	2300 F	Yes (F)
Davey [127]	2016	UK	Marines		P	1082 M	Yes (M)
Givon [61]	2000	Israel	n/a		P	2306 M	Yes (M)
Sanchez-Santos [65]	2017	UK	Marines	Recruits	P	1082 M	No (M)

*BCT* basis combat training; *n/a* not available; *R* retrospective study; *P* prospective study; *M* male; *F* female; *(M)* risk factor only for males; *(F)* risk factor only for females; *NSAID* non-steroidal anti-inflammatory drugs

*Risk factor for musculoskeletal injuries (MSkI); ^#^Deployment; ^##^Only for fractures

There is weak scientific evidence for current illness as a non-modifiable risk factor.

#### The prescription of contraceptives

Four studies focused on the prescription of contraceptives as a risk factor for MSkIs (Table 2). Most of the research was conducted within different branches of the US Armed Forces (3 studies). An additional study was conducted within the IDF. The sizes of the study populations ranged from 350 to 2962 participants. None of the four studies identified the prescription of contraceptives as a risk factor for MSkIs.

There is no scientific evidence for the prescription of contraceptives as a modifiable risk factor for MSkIs.

#### The prescription of non-steroidal anti-inflammatory drugs (NSAIDs)

Only one study focused on the prescription of a NSAID as a risk factor for MSkIs (Table 2). This study was conducted within the US Army. In this retrospective study, with 120,730 participants, the prescription of a NSAID was identified as a risk factor for MSkIs (specifically stress fractures). There may be a bias between NSAID use and increased risk for a stress fracture because with the medication, soldiers may have stayed in training longer and consequently were more likely to suffer a fracture. Therefore, this study also explored the relationship with a subset who were taking NSAIDs for non-pain or injury reasons and found a similar relationship with increased risk for MSkIs.

There is weak scientific evidence for prescription for a NSAID as a modifiable risk factor.

#### Previous MSkIs

Thirty studies focused on previous MSkIs as a risk factor for MSkIs (Table 2). Most of the research was conducted within different branches of the US Armed Forces (18 studies); the remaining research was conducted within the militaries of the UK (3 studies), Israel and China (2 studies from each), Australia, Finland, Slovenia, Sweden, and Switzerland (1 study from each nation). The sizes of the study populations ranged from 53 to 83,323 participants. Nineteen of the 30 studies identified an earlier MSkI as a risk factor for MSkIs; 7 studies did not find a significant association. Two studies found a significant association only for one sex but not the other. The remaining two studies found that an earlier MSkI reduced the risk for MSkIs.

There is strong scientific evidence for earlier MSkIs as a non-modifiable risk factor for MSkIs.

#### Prior pregnancy

Only one study focused on prior pregnancy as a risk factor for MSkIs (Table 2). This study was conducted within the US Army. In this study, with 920 female participants, prior pregnancy > 7 months prior was identified as a risk factor for MSkIs.

There is weak scientific evidence for prior pregnancy as a non-modifiable risk factor.

#### Serum iron/serum ferritin (lower)

Two studies focused on serum iron/serum ferritin as a risk factor for MSkIs (Table 2). Both studies were conducted within the IDF. The sizes of the study populations were 227 and 438 participants. Both studies identified low serum iron/serum ferritin as a risk factor for MSkIs.

There is weak scientific evidence for low serum iron/serum ferritin as a modifiable risk factor.

#### Vitamin D status [low level of 25(OH)D]

Four studies focused on vitamin D status as a risk factor for MSkIs (Table 2). The studies were conducted within the militaries of the UK (2 studies), Israel, and the US (1 study from each country). The sizes of the populations of both UK studies [65, 127] were the same. The study populations ranged from 1082 to 2306 participants. Three studies identified low vitamin D status as a risk factor for MSkIs, while another study did not find a significant association. The two studies from the UK reported different outcomes. Davey et al. [127] reported a significant difference in vitamin D level for participants who have suffered a stress fracture when compared to a group that did not [(64.2 ± 28.2) nmol/L for participants with stress fracture vs. (78.6 ± 35.9) nmol/L for participants without a stress fracture, *P* = 0.004]. Alternatively, Sanchez-Santos et al. [65] presented the results as odds ratios with a cutoff value for a low level of vitamin D at 50 nmol/L. They found no difference in the likelihood of stress fractures between the groups above and below the vitamin D level cutoff (*P* = 0.077).

In a meta-analysis by Dao et al. [23], it was reported that the mean serum 25(OH)D level was lower in stress fracture cases than in controls at the time of entry into basic training. The mean serum 25(OH)D level was also lower in the stress fracture cases at the time of stress fracture diagnosis.

There is moderate scientific evidence for a low level of vitamin D status as a modifiable risk factor.

### Occupational factors

#### Branch

Three studies focused on membership in different branches as a risk factor for MSkIs (Table 3). Two studies were conducted within the US Armed Forces and 1 within the Army of Finland. The sizes of the study populations ranged from 982 to 423,581 participants. All 3 studies identified membership to different branches as a risk factor for MSkIs.

**Table 3:** Summary of studies that focused on branch, length of service, load carriage, MOS, previous deployment, and status (active vs. reserve) as risk factors for MskIs

Study	Publication year	Country	Branches	Unit/training	Study type	*n*	Risk factor^*^
Branch							
Cameron [44]	2010	USA	Multiple	Active duty	R	423,581	Yes
Owens [128]	2009	USA	Army, Marines, Navy, Air Force	Active duty	R	19,730	Yes
Taanila [59]	2012	Finland	Army	Conscripts	P	982 M	Yes (M)
Length of service							
Hill [115]	2013	USA	Army	Active duty	R	83,323	Yes
Knapik [86]	2008	USA	Army	Paratrooper training	R	1677	Yes
Kuikka [36]	2013	Finland	Army	Conscripts	R	128,584	Yes
Mattila [38]	2007	Finland	Army	Conscripts	P	149,750 M, 2345 F	No
Reynolds [98]	2000	USA	Marines	Winter mountain training	P	356	No
Schermann [129]	2018	Israel	Army	Infantry unit vs. female unit working with dogs^##^	R	7949	Yes
Scott [122]	2015	USA	Army	Reserve Officer Training	R	165 M, 30 F	Yes
Wilkinson [60]	2009	UK	Army	Infantry	P	660	No
Load carriage							
Constantini [130]	2010	Israel	Army	Border Police Infantry	P	1423 F	Yes (F)
Knapik [83]	2013	USA	Army	Brigade Combat Team^#^	P	805	Yes
Konitzer [131]	2008	USA	n/a	Active duty^#^	R	863	Yes
Rappole [132]	2018	USA	Army	Active duty	R	368 F	No (F)
Roy [133]	2012	USA	Army	Brigade Combat Team^#^	P	246 M, 17 F	Yes
Roy [134]	2015	USA	Army	Brigade Combat Team^#^	R	536 M, 57 F	Yes
MOS							
Anderson [71]	2017	USA	Army	Light Infantry	R	4384 M, 363 F	No
Darakjy [8]	2006	USA	Army	Active duty	P	4101 M, 413 F	Yes
Roy [135]	2011	USA	Army	Brigade Combat Team	P	3066 patient encounters	Yes
Schermann [129]	2018	Israel	Army	Infantry unit vs. female unit working with dogs	R	7949	Yes
Schwartz [136]	2018	Israel	Army	Combat units	R	19,791 M	Yes (M)
Sefton [137]	2016	USA	Army	Recruits IET	P	1788 M	Yes (M)
Sharma [138]	2017	UK	Army	Recruits	P	5708	Yes
Previous deployment							
Hill [115]	2013	USA	Army	Active duty	R	83,323	Yes
Konitzer [131]	2008	USA	n/a	Active duty^#^	R	863	Yes
Roy [121]	2014	USA	Army	Active duty	R	625 F	Yes (F)
Skeehan [139]	2009	USA	Army, Marine, Navy	Active duty^#^	R	3367	No
Status (active vs. reserve)							
Canham-Chervak [52]	2006	USA	Army	Recruits	P	1156 M, 746 f	No (M) Yes (F)
Knapik [87]	2008	USA	Army	Recruits BCT	P	2147 M, 920 F	Yes (invers)
Skeehan [139]	2009	USA	Army, Marine, Navy	Active duty^#^	R	3367	Yes

*BCT* basis combat training; *IET* initial entry training; *n/a* not available; *R* retrospective study; *P* prospective study; *M* male; *F* female; *(M)* risk factor only for males; *(F)* Risk factor only for females; *MOS* Military occupational specialty

^*^Risk factor for musculoskeletal injuries (MSkI); ^#^Deployment; ^##^LOS examined in month of service

There is strong scientific evidence for branches as a non-modifiable risk factor for MSkI.

#### Length of service

Eight studies focused on the length of service as a risk factor for MSkIs (Table 3). Half of the research was conducted within different branches of the US Armed Forces (4 studies), and the remaining studies were conducted within the militaries of Finland (2 studies), Israel, and the UK (1 study from each country). The sizes of the study populations ranged from 195 to 152,095 participants. Five studies identified that military servicemen and servicewomen with a longer length of service have an increased risk for MSkIs; 3 studies did not find a significant association. Two of the largest studies only examined conscripts (Kuikka et al. [36] and Mattila et al. [38]), with a small range of lengths of service, and found conflicting results. Hill et al. [115] included a broad range of active duty personnel and showed a strong association for military servicemen and women with more than 10 years of service for an increased risk of MSkIs. Reynolds et al. [98] and Wilkinson et al. [60] detected no association, but had only a small range of lengths of service.

There is moderate scientific evidence for length of service as a non-modifiable risk factor.

#### Load carriage

Six studies focused on load carriage as a risk factor for MSkIs (Table 3). Most of the research was conducted in the US Armed Forces (5 studies); the remaining study was conducted within the IDF. The sizes of the study populations ranged from 263 to 1423 participants. Five studies identified body-borne load as a risk factor for MSkIs, with 3 of the 5 studies reporting load via self-report. One study found no association between load carriage and the risk for MSkIs.

There is strong scientific evidence for body-borne load as a modifiable risk factor for MSkI.

#### Military occupational specialty (MOS)

Seven studies focused on military occupational specialties (MOS) as a risk factor for MSkIs (Table 3). Most of the research was conducted within the US Armed Forces, 2 studies were from the IDF, and only 1 study was from the military of the UK. The sizes of the study populations ranged from 1788 to 19,791 participants. All but one study (with light infantry) identified membership in different MOSs as a risk factor for MSkIs.

There is strong scientific evidence for MOS as a non-modifiable risk factor for MSkI.

#### Previous deployment

Four studies focused on previous deployment as a risk factor for MSkIs (Table 3). All 4 studies were conducted within different branches of the US Armed Forces. The sizes of the study populations ranged from 625 to 83,323 participants. Three of the 4 studies identified previous deployment as a risk factor for MSkI, and 1 study did not find a significant association.

There is moderate scientific evidence for previous deployment as a non-modifiable risk factor.

#### Status (active *vs.* reserve)

Three studies focused on status (active vs. reserve) as a risk factor for MSkIs (Table 3). All 3 studies were conducted within the US Armed Forces. The sizes of the study populations ranged from 1902 to 3367 participants. All 3 studies identified status as a risk factor for MSkIs: 1 study only for women (when they are in the reserve instead of active duty), 1 for active personnel vs. reserve, and 1 for reserve vs. active personnel.

There is no scientific evidence for being part of the reserve (instead of active duty) as a non-modifiable risk factor for MSkIs.

### Physiological factors

#### Age

Sixty-five studies focused on age as a risk factor for MSkIs (Table 4). Most of the research was conducted within different branches of the US Armed Forces, 8 within the military of the UK, and 7 within the military of Finland; the other studies were conducted within the militaries of China (3 studies), Israel (2 studies), Belgium, Greece, Iran, Poland, and Switzerland (1 study for each country). The study populations ranged from 44 to 5,580,875 participants. Thirty-three of the 65 studies identified older age as a risk factor for MSkIs (however, the definitions of older age differ across studies); 30 studies did not find a significant association between age and MSkIs, while 1 study found a significant rise in MSkIs for younger participants when compared to older participants. When only studies with a population of 1400 or more participants were taken into account (this represents 31 of the 65 studies), 23 studies revealed a significant association between age and an increased risk for MSkIs compared to only 8 studies that did not find a significant association. When only studies that had 5000 participants or more were considered, the relationship was 12 (significant association) vs. 1 (no association).

**Table 4:** Summary of studies that focused on age, ankle dorsiflexion, and balance as risk factors for MskI

Study	Publication year	Country	Branches	Unit/training	Study type	*n*	Risk factor^*^
Age							
Anderson [70]	2015	USA	Army	Light Infantry Brigade	R	2101	Yes
Anderson [71]	2017	USA	Army	Light Infantry	R	4384 M, 363 F	Yes
Beck [140]	2000	USA	Marines		P	624 M, 693 F	No
Bedno [72]	2013	USA	Army	IET	P	8456 M	Yes (M)
Cameron [44]	2010	USA	Multiple	Active duty	R	423,581	Yes
Canham-Chervak [141]	2000	USA	Army	Recruits BCT	P	655 M, 498 F	No
Canham-Chervak [52]	2006	USA	Army	Recruits	P	1156 M, 746 F	No
Cosio-Lima [54]	2013	USA	Army	Sergeants Major Academy	R	149	No
Cowan [74]	2012	USA	Army	Trainees	P	1568 F	No (F)
Cowan [75]	2011	USA	Army	Recruits	P	7323	Yes
Craig [40]	2000	USA	Army	Airborne Division	R	242,949 aircraft exists	Yes (30 years +)
Davey [76]	2015	UK	Marines		P	1090 M	No (M)
Dixon [142]	2019	UK	Marines	Recruits	P	1065	Yes (younger)
Grier [78]	2017	USA	Army	Infantry Brigade	R	4236 M	Yes (M)
Grier [79]	2010	USA	Multiple		R	24,177 M	Yes (M)
Havenetidis [143]	2011	Greece	n/a	Recruits	P	253	Yes
Henderson [144]	2000	USA	Army	Combat medic	P	439 M, 287 F	Yes
Hill [115]	2013	USA	Army	Active duty	R	83,323	Yes
Knapik [47]	2012	USA	Army	Recruits BCT	R	475,745 M, 107,906 F	Yes
Knapik [145]	2006	USA	Army	Recruits BCT	P	1174 M, 898 F	Yes
Knapik [82]	2013	USA	Army	Army military police training	P	1838 M, 553 F	Yes
Knapik [146]	2007	USA	Army	Mechanics	R	518 M, 43 F	No
Knapik [84]	2007	USA	Army	Band	R	159 M, 46 F	No
Knapik [85]	2001	USA	Army	Recruits	P	182 M, 168 F	No
Knapik [86]	2008	USA	Army	Paratrooper training	R	1677	Yes
Knapik [87]	2008	USA	Army	Recruits BCT	P	2147 M, 920 F	Yes
Knapik [88]	2009	USA	Marines	Recruits BCT	P	840 M, 571 F	No
Korvala [89]	2010	Finland	n/a	Conscripts	P	192	Yes
Kuikka [36]	2013	Finland	Army	Conscripts	R	128,584	Yes
Lappe [55]	2005	USA	Army	Recruits BCT	R	4139 F	Yes (F)
Lappe [56]	2001	USA	Army	Recruits BCT	P	3758 F	Yes (F)
Lauder [90]	2000	USA	Army	Active duty	P	230 F	No (F)
Ma [147]	2016	China	n/a		R	2479	No
Mahieu [148]	2006	Belgium	n/a	Recruits Royal Military Academy	P	69 M	No (M)
Mattila [38]	2007	Finland	Army	Conscripts	P	149,750 M, 2345 F	Yes
Moran [149]	2013	Israel	Army	Recruits	P	44	No
Munnoch [91]	2007	UK	Marines		P	1115 M	Yes (M)
Nunns [150]	2016	UK	Marines	Recruits	P	160 M	No (M)
Nye [151]	2016	USA	Air Force	Recruits BCT	R	67,525	Yes
Owens [152]	2007	USA	n/a	Active duty	R	4451	Yes
Owens [128]	2009	USA	Army, Marines, Navy, Air Force	Active duty	R	19,730	Yes
Parr [153]	2015	USA	Army	Special Operations Forces	P	106	No
Pihlajamäki [93]	2019	Finland	n/a	Full duty	R	4029 M	No (M)
Rabin [154]	2014	Israel	Army	Recruits	P	70 M	No (M)
Reynolds [96]	2009	USA	Army	Infantry	P	181	No
Reynolds [97]	2002	USA	Army	Construction engineers & Combat artillery soldiers	P	313	No
Roos [99]	2015	Switzerland	Army	Recruits	P	651 M	No (M)
Roy [133]	2012	USA	Army	Brigade Combat Team^#^	P	246 M, 17 F	No
Roy [121]	2014	USA	Army	Active duty	R	625 F	Yes (F)
Ruohola [100]	2006	Finland	n/a	Recruits	P	756 M	No (M)
Sanchez-Santos [65]	2017	UK	Marines	Recruits	P	1082 M	Yes (M)
Schneider [58]	2000	USA	Army	Airborne Div	R	1214	Yes
Sefton [137]	2016	USA	Army	Recruits IET	P	1788 M	Yes (M)
Shaffer [109]	2006	USA	Marines	Recruits BCT	R	2962 F	No (F)
Sharma [102]	2019	UK	Army	Infantry recruits	P	562 M	No (M)
Sharma [103]	2011	UK	Army	Infantry recruits	P	468 M	No (M)
Skeehan [139]	2009	USA	Army, Marine, Navy	Active duty^#^	R	3367	No
Sobhani [155]	2015	Iran	n/a	Recruits	R	181 M	No (M)
Sormaala [39]	2006	Finland	n/a	Recruits	R	118,149	No
Taanila [59]	2012	Finland	Army	Conscripts	P	982 M	Yes (M)
Trybulec [156]	2016	Poland	Army	Airborne Brigade	R	162 M, 3 F	Yes
Wang [106]	2003	China	n/a	Military Police Forces Training	R	805 M	No (M)
Waterman [31]	2016	USA	Multiple	Active Duty	R	5,580,875	Yes
Wilkinson [60]	2009	UK	Army	Infantry	P	660	Yes
Zhao [108]	2016	China	Army	Recruits	P	1398 M	No (M)
Ankle dorsiflexion (limited)							
Dixon [30]	2006	UK	Marines	Recruits	R	20	No
Rabin [154]	2014	Israel	Army	Recruits	P	70 M	No (M)
Balance (low)							
Heebner [157]	2017	USA	Army	Special Operation Forces	P	95	No
Sell [158]	2014	USA	Special Operation Forces		P	226	Yes

*BCT* basis combat training; *IET* initial entry training; *n/a* not available; *R* retrospective study; *P* prospective study; *M* male; *F* female; *(M)* risk factor only for males; *(F)* risk factor only for females

*Risk factor for musculoskeletal injuries (MSkI); ^#^Deployment

There is moderate scientific evidence for age as a non-modifiable risk factor.

#### Ankle dorsiflexion (limited)

Only 2 studies focused on limited ankle dorsiflexion as a risk factor for MSkIs (Table 4). One study was conducted within the IDF, and one in the armed forces of the UK. The sizes of the study populations were 20 and 70 participants, respectively. In both studies, limited ankle dorsiflexion was not significantly identified as a risk factor for MSkIs.

There is no scientific evidence for limited ankle dorsiflexion as a non-modifiable risk factor.

#### Balance (low)

Two studies focused on low balance as a risk factor for MSkIs (Table 4). These studies were conducted within the special operations forces of the US military. In the larger study, poor balance (measured as single-leg balance with the eyes open, and the eyes closed on a force plate) was identified as a risk factor for MSkIs, whereas in the other studies, no association was identified.

There is weak scientific evidence for low balance as a modifiable risk factor.

#### BMI: in general

Fifty-two studies focused on BMI (in general) as a risk factor for MSkIs (Table 5). BMI in general means that the studies have looked at BMI without categorization (such as obese, overweight, underweight categories). This makes it very difficult to compare different study outcomes. Most of the research was conducted within different branches of the US Armed Forces (24 studies); 9 studies within the military of the UK, 6 within the Finnish armed forces, and 5 within the IDF. The remaining studies were conducted in the militaries of Switzerland (3 studies), Greece (2 studies), Australia, Belgium, and Malta (1 study each). The sizes of the study populations ranged from 44 to 238,772 participants. Fourteen of the 52 studies identified BMI as a risk factor for MSkIs. Thirteen studies found that higher BMI was a risk factor; 1 study found that lower BMI was a risk factor. Thirty-five studies did not find a significant association between BMI and MSkIs, and 3 studies found that BMI is a risk factor for men, but not for women.

**Table 5:** Summary of studies that focused on BMI (in general), obesity, being overweight, and being underweight as risk factors for MskIs

Study	Publication year	Country	Branches	Unit/training	Study type	*n*	Risk factor^*^
BMI (in general)							
Allsopp [159]	2003	UK	Navy	Recruits	R	1287 M, 354 F	Yes
Beck [140]	2000	USA	Marines		P	624 M, 693 F	Yes (M), no (F)
Bedno [35]	2019	USA	Army	Recruits BCT	R	238,772	Yes (M), no (F)
Billings [160]	2004	USA	Air Force	Recruits BCT	R	2006	Yes
Blacker [161]	2008	UK	Army	Recruits	R	11,937 M, 1480 F	Yes
Burgi [126]	2011	USA	Navy	Recruits	P	2300 F	No (F)
Cosio-Lima [54]	2013	USA	Army	Sergeants Major Academy	R	149	No
Davey [76]	2015	UK	Marines		P	1090 M	No (M)
Garnock [113]	2018	Australia	Navy	Recruits	P	95 M, 39 F	No
George [114]	2012	USA	Army	Combat medics	P	1230	Yes
Havenetidis [162]	2017	Greece	Army	Officer recruits	P	268 M	No (M)
Havenetidis [143]	2011	Greece	n/a	Recruits	P	253	No
Jones [34]	2017	USA	Army	Recruits BCT	R	143,398 M, 41,727 F	Yes
Knapik [145]	2006	USA	Army	Recruits BCT	P	1174 M, 898 F	No
Knapik [82]	2013	USA	Army	Army military police training	P	1838 M, 553 F	Yes
Knapik [146]	2007	USA	Army	Mechanics	R	518 M, 43 F	Yes (M)
Knapik [84]	2007	USA	Army	Band	R	159 M, 46 F	No
Knapik [85]	2001	USA	Army	Recruits	P	182 M, 168 F	No
Knapik [86]	2008	USA	Army	Paratrooper training	R	1677	No
Knapik [87]	2008	USA	Army	Recruits BCT	P	2147 M, 920 F	No
Knapik [88]	2009	USA	Marines	Recruits BCT	P	840 M, 571 F	No
Kodesh [163]	2015	Israel	n/a	Combat Fitness Instructor Course	P	158 F	No
Korvala [89]	2010	Finland	n/a	Conscripts	P	192	Yes
Kupferer [164]	2014	USA	Air Force	Trainees	R	141	No
Lauder [90]	2000	USA	Army	Active duty	P	230 F	Yes (F)
Mahieu [148]	2006	Belgium	n/a	Recruits Royal Military Academy	P	69 M	No
Mattila [38]	2007	Finland	Army	Conscripts	P	149,750 M, 2345 F	No
Moran [149]	2013	Israel	Army	Recruits	P	44	No
Moran [63]	2012	Israel	Army	Elite combat unit BCT	P	74	No (M)
Moran [125]	2008	Israel	Army	Recruits	P	227 F	Yes (F)
Munnoch [91]	2007	UK	Marines		P	1115 M	No (M)
Nunns [150]	2016	UK	Marines	Recruits	P	160 M	Yes (M)
Nye [151]	2016	USA	Air Force	Recruits BCT	R	67,525	No
Parr [153]	2015	USA	Army	Special Operations Forces	P	106	No
Pihlajamäki [93]	2019	Finland	n/a	Full duty	R	4029 M	No (M)
Psaila [94]	2017	Malta	n/a	Recruits BCT	P	114 M, 13 F	No
Rabin [154]	2014	Israel	Army	Recruits	P	70 M	No (M)
Rappole [95]	2017	USA	Army	Army Brigade	R	1099	Yes
Reynolds [98]	2000	USA	Marines	Winter mountain training	P	356	No
Rice [120]	2017	UK	Marines	Recruits	P	147 M	Yes (M, especially lower BMI)
Roos [99]	2015	Switzerland	Army	Recruits	P	651 M	No (M)
Ruohola [100]	2006	Finland	n/a	Recruits	P	756 M	No (M)
Scott [122]	2015	USA	Army	Reserve Officer Training	R	165 M, 30 F	No
Shaffer [109]	2006	USA	Marines	Recruits BCT	R	2962 F	No (F)
Sharma [102]	2019	UK	Army	Infantry recruits	P	562 M	No (M)
Sharma [103]	2011	UK	Army	Infantry recruits	P	468 M	No (M)
Sillanpää [51]	2008	Finland	n/a	Conscripts	R	128,508 M	No (M)
Sormaala [39]	2006	Finland	n/a	Recruits	R	118,149	No
Waterman [165]	2010	USA	Military Academy		R	10,511 person years	Yes (M), no (F)
Wilkinson [60]	2009	UK	Army	Infantry	P	660	No
Wunderlin [107]	2015	Switzerland	Army	Recruits	P	230 M	Yes (M)
Wyss [68]	2014	Switzerland	Army	Recruits BCT	P	1676	NO
Obesity (BMI ≥ 30 kg/m^2^)							
Anderson [70]	2015	USA	Army	Light Infantry Brigade	R	2101	Yes
AMSA [43]	2000	USA	Army	Active duty	R	387,536	Yes
Bedno [72]	2013	USA	Army	IET	P	8456 M	Yes (M)
Billings [160]	2004	USA	Air Force	Recruits BCT	R	2006	Yes
Canham-Chervak [52]	2006	USA	Army	Recruits	P	1156 M, 746 F	Yes
Cowan [74]	2012	USA	Army	Trainees	P	1568 F	No (F)
Cowan [75]	2011	USA	Army	Recruits	P	7323	Yes
Gundlach [166]	2012	Germany	Army	Active duty	P	410	Yes
Henderson [144]	2000	USA	Army	Combat medic	P	439 M, 287 F	Yes
Hruby [48]	2016	USA	Army		R	736,608	Yes
Jones [34]	2017	USA	Army	Recruits BCT	R	143,398 M, 41,727 F	Yes
Kuikka [36]	2013	Finland	Army	Conscripts	R	128,584	Yes
Ma [147]	2016	China	n/a		R	2479	Yes
Packnett [41]	2011	USA	Army	Recruits BCT	R	217,468 M, 47,813 F	Yes
Rappole [95]	2017	USA	Army	Army Brigade	R	1099	Yes
Taanila [123]	2010	Finland	n/a	Conscripts	P	944 M	Yes (M)
Taanila [59]	2012	Finland	Army	Conscripts	P	982 M	Yes (M)
Overweight (BMI ≥ 25 and < 30 kg/m^2^)							
Anderson [70]	2015	USA	Army	Light Infantry Brigade	R	2101	Yes
Bedno [72]	2013	USA	Army	IET	P	8456 M	No (M)
Billings [160]	2004	USA	Air Force	Recruits BCT	R	2006	Yes
Canham-Chervak [52]	2006	USA	Army	Recruits BCT	P	1156 M, 746 F	Yes
Cowan [74]	2012	USA	Army	Trainees	P	1568 F	No (F)
Grier [78]	2017	USA	Army	Infantry Brigade	R	4236 M	Yes (M)
Gundlach [166]	2012	Germany	Army	Active duty	P	410	Yes
Henderson [144]	2000	USA	Army	Combat medic	P	439 M, 287 F	Yes
Hruby [48]	2016	USA	Army		R	736,608	Yes
Knapik [47]	2012	USA	Army	Recruits BCT	R	475,745 M, 107,906 F	Yes (M), no (F)
Kuikka [36]	2013	Finland	Army	Conscripts	R	128,584	No
Ma [147]	2016	China	n/a		R	2479	Yes
Mattila [37]	2007	Finland	n/a	Conscripts	R	133,943 M, 2044 F	Yes
Rappole [95]	2017	USA	Army	Army Brigade	R	1099 M	Yes (M)
Taanila [123]	2010	Finland	n/a	Conscripts	P	944 M	Yes (M)
Taanila [59]	2012	Finland	Army	Conscripts	P	982 M	No (M)
Underweight (BMI < 18.5 kg/m^2^)							
AMSA [43]	2000	USA	Army	Active duty	R	387,536	Yes
Bedno [72]	2013	USA	Army	IET	P	8456 M	Yes (M)
Billings [160]	2004	USA	Air Force	Recruits BCT	R	2006	Yes
Cowan [74]	2012	USA	Army	Trainees	P	1568 F	No (F)
Finestone [167]	2008	Israel	Army	Light Infantry training	P	36 M, 99 F	Yes
Grier [78]	2017	USA	Army	Infantry brigade	R	4236 M	Yes (M)
Hruby [48]	2016	USA	Army		R	736,608	Yes
Jones [34]	2017	USA	Army	Recruits BCT	R	143,398 M, 41,727 F	Yes
Knapik [47]	2012	USA	Army	Recruits BCT	R	475,745 M, 107,906 F	Yes
Kuikka [36]	2013	Finland	Army	Conscripts	R	128,584	No
Packnett [41]	2011	USA	Army	Recruits BCT	R	217,468 M, 47,813 F	Yes
Reynolds [96]	2009	USA	Army	Infantry	P	181	Yes
Taanila [104]	2015	Finland	Army	Conscripts	P	1411 M	Yes (M)
Taanila [59]	2012	Finland	Army	Conscripts	P	982 M	No (M)
Wang [106]	2003	China	n/a	Military Police Forces Training	R	805 M	Yes (M)

*BMI* body mass index; *BCT* basis combat training; *IET* initial entry training; *n/a* not available; *R* Retrospective study; *P* prospective study; *M* male; *F* female; *(M)* risk factor only for males; *(F)* risk factor only for females

*Risk factor for musculoskeletal injuries (MSkI)

There is insufficient scientific evidence for BMI in general as a modifiable risk factor.

#### BMI: obesity (BMI ≥ 30 kg/m^2^)

Seventeen studies focused on obesity as a risk factor for MSkIs (Table 5). Most of the research was conducted within different branches of the US Armed Forces (12 studies). Additional studies were conducted within the militaries of Finland (3 studies), China, and Germany (1 study for each country). The sizes of the study populations ranged from 410 to 387,536 participants. Sixteen studies identified obesity as a risk factor for MSkIs; only one study, with 1568 participants, did not find a significant association.

There is strong scientific evidence for obesity (BMI ≥ 30 kg/m^2^) as a modifiable risk factor for MSkIs.

#### BMI: overweight (BMI ≥ 25 and < 30 kg/m^2^)

Sixteen studies focused on being overweight as a risk factor for MSkIs (Table 5). Most of the research was conducted within different branches of the US Armed Forces (10 studies); the remaining studies were conducted within the Finnish armed forces (4 studies) and within the militaries of China and Germany (1 study each). The sizes of the study populations ranged from 410 to 736,608 participants. Eleven studies identified being overweight as a risk factor for MSkIs; 4 studies did not find a significant association. One study found an association for men but not for women. It is important to acknowledge that these findings are based on BMI alone; none of the 16 studies analyzed the body composition of the included soldiers in detail (i.e., body fat or muscle mass).

There is strong scientific evidence for being overweight (BMI ≥ 25 and < 30 kg/m^2^) as a modifiable risk factor for MSkI.

#### BMI: underweight (BMI < 18.5 kg/m^2^)

Fifteen studies focused on being underweight as a risk factor for MSkIs (Table 5). Most of the research was conducted within different branches of the US Armed Forces (10 studies); the remaining studies were conducted within the militaries of Finland (3 studies), China, and Israel (1 study each). The sizes of the study populations ranged from 135 to 736,608 participants. Twelve studies identified being underweight as a risk factor for MSkIs, and 3 studies did not find a significant association.

There is strong scientific evidence for being underweight (BMI < 18.5 kg/m^2^) as a modifiable risk factor for MSkIs.

#### Body fat (higher)

Eight studies focused on body fat as a risk factor for MSkIs (Table 6). The research was conducted within the armies of Greece (2 studies), Iran (1 study), Israel (2 studies), and the US (3 studies); the studies included different methods for measuring body fat (e.g., self-report, circumference, dual-energy X-ray absorptiometry, 4-site skinfold test). The sizes of the study populations ranged from 44 to 583,651 participants. Six of the 8 studies identified a higher percentage of body fat as a risk factor for MSkIs, and 2 studies did not find a significant association. A retrospective study by Knapik et al. [46], with more than a half million participants, showed a relationship between a greater percentage of body fat and a higher risk for MSkIs.

**Table 6:** Summary of studies that focused on body fat, body height, and body weight as risk factors for MskIs

Study	Publication year	Country	Branches	Unit/training	Study type	*n*	Risk factor^*^
Body fat (higher)							
Anderson [71]	2017	USA	Army	Light Infantry	R	4384 M, 363 F	Yes
Havenetidis [162]	2017	Greece	Army	Officer recruits	P	268 M	Yes (M)
Havenetidis [143]	2011	Greece	n/a	Recruits	P	253	Yes
Knapik [46]	2018	USA	Army	Recruits BCT	R	475,745 M, 107,906 F	Yes
Kodesh [163]	2015	Israel	n/a	Combat Fitness Instructor Course	P	158 F	Yes (F)
Krauss [168]	2017	USA	Army	Recruits BCT	R	1900 F	Yes (F)
Moran [149]	2013	Israel	Army	Recruits	P	44	No
Sobhani [155]	2015	Iran	n/a	Recruits	R	181 M	No (M)
Body height (higher)							
Beck [140]	2000	USA	Marines		P	624 M, 693 F	Yes (M), no (F)
Blacker [161]	2008	UK	Army	Recruits	R	11,937 M, 1480 F	No
Cosio-Lima [54]	2013	USA	Army	Sergeants Major Academy	R	149	No
Davey [76]	2015	UK	Marines		P	1090 M	No (M)
Fallowfield [77]	2018	UK	Air Force	Recruits	P	990 M, 203 F	Yes
Finestone [112]	2011	Israel	Army	Elite infantry soldier	P	77 M	No (M)
Givon [61]	2000	Israel	n/a		P	2306 M	No (M)
Kelly [80]	2000	USA	Navy	Recruits BCT	R	86 F	Yes (F)
Knapik [47]	2012	USA	Army	Recruits BCT	R	475,745 M, 107,906 F	Yes
Knapik [145]	2006	USA	Army	Recruits BCT	P	1174 M, 898 F	No
Knapik [146]	2007	USA	Army	Mechanics	R	518 M, 43 F	No
Knapik [84]	2007	USA	Army	Band	R	159 M, 46 F	No
Knapik [86]	2008	USA	Army	Paratrooper training	R	1677	No
Knapik [87]	2008	USA	Army	Recruits BCT	P	2147 M, 920 F	No
Knapik [88]	2009	USA	Marines	Recruits BCT	P	840 M, 571 F	No
Kodesh [163]	2015	Israel	n/a	Combat Fitness Instructor Course	P	158 F	No
Korvala [89]	2010	Finland	n/a	Conscripts	P	192	Yes
Kuikka [36]	2013	Finland	Army	Conscripts	R	128,584	No
Lappe [56]	2001	USA	Army	Recruits BCT	P	3758 F	No (F)
Ma [147]	2016	China	n/a		R	2479	No
Mahieu [148]	2006	Belgium	n/a	Recruits Royal Military Academy	P	69 M	No (M)
Mattila [38]	2007	Finland	Army	Conscripts	P	149,750 M, 2345 F	No
Monnier [119]	2019	Sweden	Marines	Training course	P	48 M, 5 F	Yes
Moran [149]	2013	Israel	Army	Recruits	P	44	No
Moran [63]	2012	Israel	Army	Elite combat unit BCT	P	74	No
Moran [125]	2008	Israel	Army	Recruits	P	227 F	Yes (F)
Munnoch [91]	2007	UK	Marines		P	1115 M	No (M)
Nunns [150]	2016	UK	Marines	Recruits	P	160 M	No (M)
Parr [153]	2015	USA	Army	Special Operations Forces	P	106	No
Reynolds [96]	2009	USA	Army	Infantry	P	181	No
Reynolds [97]	2002	USA	Army	Construction engineers & Combat artillery soldiers	P	313	Yes (to be shorter)
Reynolds [98]	2000	USA	Marines	Winter mountain training	P	356	No
Ruohola [100]	2006	Finland	n/a	Recruits	P	756 M	No (M)
Shaffer [109]	2006	USA	Marines	Recruits BCT	R	2962 F	No (F)
Sharma [102]	2019	UK	Army	Infantry recruits	P	562 M	No (M)
Sharma [103]	2011	UK	Army	Infantry recruits	P	468 M	No (M)
Sillanpää [51]	2008	Finland	n/a	Conscripts	R	128,508 M	Yes (M)
Sobhani [155]	2015	Iran	n/a	Recruits	R	181 M	No (M)
Sormaala [39]	2006	Finland	n/a	Recruits	R	118,149	No
Sulsky [42]	2018	USA	Army	Recruits BCT	R	278,045 M, 55,302 F	Yes
Taanila [59]	2012	Finland	Army	Conscripts	P	982 M	No (M)
Trybulec [156]	2016	Poland	Army	Airborne Brigade	R	162 M, 3 F	No
Wang [106]	2003	China	n/a	Military Police Forces Training	R	805 M	No (M)
Waterman [165]	2010	USA	Military Academy		R	10,511 person years	Yes
Wilkinson [60]	2009	UK	Army	Infantry	P	660	No
Zhao [108]	2016	China	Army	Recruits	P	1398 M	No (M)
Body weight (higher)							
Beck [140]	2000	USA	Marines		P	624 M, 693 F	Yes (M), no (F)
Blacker [161]	2008	UK	Army	Recruits	R	11,937 M, 1480 F	No
Davey [76]	2015	UK	Marines		P	1090 M	No (M)
Davey [127]	2016	UK	Marines		P	1082 M	No (M)
Finestone [112]	2011	Israel	Army	Elite infantry soldier	P	77 M	No (M)
Givon [61]	2000	Israel	n/a		P	2306 M	Yes (M)
Havenetidis [162]	2017	Greece	Army	Officer recruits	P	268 M	Yes (M)
Hughes [169]	2008	Australia	Special Operation Forces	Active duty	R	554 descents	Yes
Kelly [80]	2000	USA	Navy	Recruits BCT	R	86 F	Yes (F)
Knapik [47]	2012	USA	Army	Recruits BCT	R	475,745 M, 107,906 F	Yes (invers)
Knapik [145]	2006	USA	Army	Recruits BCT	P	1174 M, 898 F	No
Knapik [146]	2007	USA	Army	Mechanics	R	518 M, 43 F	Yes
Knapik [84]	2007	USA	Army	Band	R	159 M, 46 F	No
Knapik [86]	2008	USA	Army	Paratrooper training	R	1677	Yes
Knapik [87]	2008	USA	Army	Recruits BCT	P	2147 M, 920 F	No
Knapik [88]	2009	USA	Marines	Recruits BCT	P	840 M, 571 F	No (M), yes (F)
Kodesh [163]	2015	Israel	n/a	Combat Fitness Instructor Course	P	158 F	No (F)
Korvala [89]	2010	Finland	n/a	Conscripts	P	192	Yes
Lappe [56]	2001	USA	Army	Recruits BCT	P	3758 F	Yes (F)
Ma [147]	2016	China	n/a		R	2479	No
Mahieu [148]	2006	Belgium	n/a	Recruits Royal Military Academy	P	69 M	No (M)
Moran [149]	2013	Israel	Army	Recruits	P	44	No
Moran [63]	2012	Israel	Army	Elite combat unit BCT	P	74	No
Monnier [119]	2019	Sweden	Marines	Training course	P	48 M, 5 F	No
Munnoch [91]	2007	UK	Marines		P	1115 M	No (M)
Nunns [150]	2016	UK	Marines	Recruits	P	160 M	No (M)
Parr [153]	2015	USA	Army	Special Operations Forces	P	106	No
Reynolds [96]	2009	USA	Army	Infantry	P	181	No
Reynolds [97]	2002	USA	Army	Construction engineers & Combat artillery soldiers	P	313	Yes
Reynolds [98]	2000	USA	Marines	Winter mountain training	P	356	No
Rice [120]	2017	UK	Marines	Recruits	P	147 M	Yes (M) (invers)
Robinson [57]	2016	UK	Army	Recruits	P	1810	Yes
Ruohola [100]	2006	Finland	n/a	Recruits	P	756 M	No (M)
Sanchez-Santos [65]	2017	UK	Marines	Recruits	P	1082 M	Yes (M) (invers)
Schermann [129]	2018	Israel	Army	Infantry unit vs. female unit working with dogs	R	7949	Yes
Shaffer [109]	2006	USA	Marines	Recruits BCT	R	2962 F	No (F)
Sharma [102]	2019	UK	Army	Infantry recruits	P	562 M	No (M)
Sharma [103]	2011	UK	Army	Infantry recruits	P	468 M	No (M)
Sillanpää [51]	2008	Finland	n/a	Conscripts	R	128,508 M	Yes (M)
Sobhani [155]	2015	Iran	n/a	Recruits	R	181 M	No (M)
Sormaala [39]	2006	Finland	n/a	Recruits	R	118,149	No
Trybulec [156]	2016	Poland	Army	Airborne Brigade	R	162 M, 3 F	No
Waterman [165]	2010	USA	Military Academy		R	10,511 person years	Yes
Wilkinson [60]	2009	UK	Army	Infantry	P	660	No
Zhao [108]	2016	China	Army	Recruits	P	1398 M	No (M)

*BCT* basis combat training; *n/a* not available; *R* retrospective study; *P* prospective study; *M* male; *F* female; *(M)* risk factor only for males; *(F)* risk factor only for females

*Risk factor for musculoskeletal injuries (MSkI)

There is strong scientific evidence for higher body fat as a modifiable risk factor for MSkIs.

#### Body height (higher)

Forty-six studies focused on body height as a risk factor for MSkIs (Table 6). Most of the research was conducted within different branches of the US Armed Forces (18 studies); 8 within the military of the UK, 7 within the military of Finland, and 6 studies within the IDF; the other studies were conducted within the military of China (3 studies), Belgium, Iran, Poland, and Sweden (1 study each). The sizes of the study populations ranged from 44 to 583,651 participants. Eight of the 46 studies identified a taller stature as a risk factor for MSkIs, and 35 studies did not find a significant association. One study found a significant increase in MSkIs associated with a taller stature for men but not for women, and one study found that a shorter stature was a significant risk factor for MSkIs.

There is insufficient scientific evidence for body height as a non-modifiable risk factor for MSkIs.

#### Body weight (higher)

Forty-five studies focused on body weight as a risk factor for MSkIs (Table 6). Most of the research was conducted within different branches of the US Armed Forces (16 studies); 11 studies within the military of the UK, and 6 within the IDF. The remaining studies were conducted within the militaries of Finland (4 studies), China (2 studies), Australia, Belgium, Greece, Iran, Poland, and Sweden (1 study each). The sizes of the study populations ranged from 44 to 583,651 participants. Thirteen of the 45 studies identified a higher body weight as a risk factor for MSkIs, 27 did not find a significant association between body weight and MSkIs, and 3 studies found a significant increase in MSkIs for a lower body weight. Two studies found different outcomes regarding the participants’ sex.

There is insufficient scientific evidence for higher body weight as a modifiable risk factor.

#### Bone (mineral) density (low)

Three studies focused on low bone (mineral) density as a risk factor for MSkIs (Table 7). All 3 studies were conducted in the US Army. The sizes of the study populations ranged from 230 to 891 participants. Two studies identified low bone (mineral) density as a risk factor for MSkIs; one study did not find a significant association.

**Table 7:** Summary of studies that focused on bone (mineral) density, external rotation of the hip, flexibility, and foot type as risk factors for MskIs

Study	Publication year	Country	Branches	Unit/training	Study type	*n*	Risk factor^*^
Bone (mineral) density (low)							
Cosman [64]	2013	USA	Army	Military Academy	P	755 M, 136 F	Yes
Knapik [85]	2001	USA	Army	Recruits	P	182 M, 168 F	No
Lauder[90]	2000	USA	Army	Active duty	P	230 F	Yes (F)
External rotation of the hip (higher)							
Burne [170]	2004	Australia	Military Academy		P	122 M, 25 F	No
Finestone [112]	2011	Israel	Army	Elite infantry soldier	P	77 M	No (M)
Garnock [113]	2018	Australia	Navy	Recruits	P	95 M, 39 F	Yes
Rauh [171]	2010	USA	Marines	Recruits BCT	P	748 F	Yes (F)
Sobhani [155]	2015	Iran	n/a	Recruits	R	181 M	Yes (M)
Flexibility (lower)							
Heebner [157]	2017	USA	Army	Special Operations Forces	P	95	No
Keenan [172]	2017	USA	Multiple	Special Forces	P	726	Yes^#,##^
Knapik [85]	2001	USA	Army	Recruits	P	182 M, 168 F	No^#^
Nagai [92]	2017	USA	Army	Airborne Div	P	275	No^###^
Wang [106]	2003	China	n/a	Military Police Forces Training	R	805 M	No (M)
Foot type							
Esterman [173]	2005	Australia	Air Force	Recruits	P	230	No
Hetsroni [174]	2006	Israel	Army	Recruits	P	405 M	No^&^
Levy [175]	2006	USA	n/a	Military Academy Cadets	R	431 M, 73 F	Yes^&&^
Nunns [150]	2016	UK	Marines	Recruits	P	160 M	Yes (M)^&&&,&&&&^
Psaila [94]	2017	Malta	n/a	Recruits BCT	P	114 M, 13 F	No
Reynolds [98]	2000	USA	Marines	Winter mountain training	P	356	Yes^&&&&&^
Rice [120]	2017	UK	Marines	Recruits	P	147 M	Yes (M)^&&&^
Yates [176]	2004	UK	Navy	Recruits	P	84 M, 40 F	Yes

*BCT* basis combat training; *n/a* not available; *R* retrospective study; *P* prospective study; *M* male; *F* female; *(M)* risk factor only for males; *(F)* risk factor only for females

*Risk factor for musculoskeletal injuries (MSkI); ^#^Hamstring-flexibility; ^##^Gastrocnemius-soleus flexibility; ^###^Several muscle groups (shoulder, trunk rotation, hip extension, active knee extension, ankle dorsiflexion, ankle plantarflexion); ^&^For any type for foot pronation; ^&&^Pes planus; ^&&&^Width malleolar; ^&&&&^Arch index, corrected calf girth; ^&&&&&^Forefoot varus

There is insufficient scientific evidence for low bone (mineral) density as a non-modifiable risk factor.

#### External rotation of the hip (higher)

Five studies focused on external rotation (range of motion) of the hip as a risk factor for MSkIs (Table 7). The research was conducted within the militaries of Australia (2 studies), Iran, Israel, and the US (each 1 study). The range of motion of the hip was measured in different ways across the identified studies. The sizes of the study populations ranged from 77 to 748 participants. Three studies (including the two with the most participants) identified that higher external rotation of the hip is a risk factor for MSkIs; two studies did not find a significant association.

There is insufficient scientific evidence for higher external rotation of the hip as a non-modifiable risk factor.

#### Flexibility (lower)

Five studies focused on flexibility at different anatomical locations as a risk factor for MSkIs (Table 7). Most of the research was conducted within different branches of the US Armed Forces (4 studies), and 1 study was conducted by armed forces from China. The sizes of the study populations ranged from 95 to 805 participants. Only 1 study identified low flexibility as a risk factor for MSkIs, and 5 studies did not find a significant association.

There is insufficient scientific evidence for lower flexibility as a modifiable risk factor.

#### Foot type

Eight studies focused on foot type (e.g., anatomic differences such as a pes planus, a wide malleolar or a forefoot varus) as a risk factor for MSkIs (Table 7). The studies were conducted within the militaries of the UK (3 studies), USA (2 studies), Australia, Israel, and Malta (1 study from each country). The sizes of the study populations ranged from 124 to 504 participants. Five studies identified different foot types as a risk factor for MSkI, while 3 studies did not.

There is moderate scientific evidence for different foot types as a non-modifiable risk factor.

#### Genetic factors

Two studies focused on genetic factors as risk factors for MSkIs (Table 8). One study was conducted within the military of China and 1 within the military of Finland. The study populations ranged from 192 to 1398 participants. Both studies identified an association between certain genetic factors and an increased risk for MSkIs. The analyzed genetic factors were different between the 2 studies, so a comparison was not possible. Korvala et al*.* [89] examined genes involved in bone metabolism and pathology, and Zhao et al. [108] looked at a specific growth differentiation factor 5 (GDF5) polymorphism between recruits with and without stress fractures.

**Table 8:** Summary of studies that focused on genetic factors, late menarche, muscular strength, and physical fitness as risk factors for MSkIs

Study	Publication year	Country	Branches	Unit/training	Study type	*n*	Risk factor^*^
Genetic factors							
Korvala [89]	2010	Finland	n/a	Conscripts	P	192	Yes
Zhao [108]	2016	China	Army	Recruits	P	1398 M	Yes (M)
Late menarche							
Cosman [64]	2013	USA	Army	Military Academy	P	136 F	Yes
Knapik [81]	2010	USA	Air Force	Recruits BCT	P	375 F	No
Knapik [87]	2008	USA	Army	Recruits BCT	P	920 F	No
Knapik [88]	2009	USA	Marines	Recruits BCT	P	571 F	No
Lappe [56]	2001	USA	Army	Recruits BCT	P	3758 F	No
Shaffer [109]	2006	USA	Marines	Recruits BCT	R	2962 F	No
Trone [105]	2014	USA	Marine Corp Air Force Army	Recruits BCT	R	597 F	Yes
Muscular strength (lower)							
Blacker [161]	2008	UK	Army	Recruits	R	11,937 M, 1480 F	No
Heebner [157]	2017	USA	Army	Special Operation Forces	P	95	Yes
Knapik [84]	2007	USA	Army	Band	R	159 M, 46 F	No
Kuikka [36]	2013	Finland	Army	Conscripts	R	128,584	Yes
Mattila [38]	2007	Finland	Army	Conscripts	P	149,750 M, 2345 F	Yes
Nagai [92]	2017	USA	Army	Airborne Div	P	275	No
Parr [153]	2015	USA	Army	Special Operations Forces	P	106	No^##^
Roy [177]	2012	USA	Army	Brigade Combat Team^#^	R	593	Yes
Ruohola [100]	2006	Finland	n/a	Recruits	P	756 M	Yes (M)
Sillanpää [51]	2008	Finland	n/a	Conscripts	R	128,508 M	No (M)
Wunderlin [107]	2015	Switzerland	Army	Recruits	P	230 M	Yes (M)
Physical fitness (low)							
Allsopp [159]	2003	UK	Navy	Recruits	R	1287 M, 354 F	Yes
Anderson [70]	2015	USA	Army	Light Infantry Brigade	R	2101	Yes
Anderson [71]	2017	USA	Army	Light Infantry	R	4384 M, 363 F	Yes
Beck [140]	2000	USA	Marines		P	624 M, 693 F	Yes
Bedno [72]	2013	USA	Army	IET	P	8456 M	Yes (M)
Bedno [35]	2019	USA	Army	Recruits BCT	R	238,772	No (M), yes (F)
Bell [27]	2000	USA	Army	Recruits	P	861	Yes
Blacker [161]	2008	UK	Army	Recruits	R	11,937 M, 1480 F	Yes
Brooks [73]	2019	USA	Army	Recruits BCT	R	1460 M, 540 F	Yes
Canham-Chervak [141]	2000	USA	Army	Recruits BCT	P	655 M, 498 F	Yes
Canham-Chervak [52]	2006	USA	Army	Recruits BCT	P	1156 M, 746 F	Yes
Cosio-Lima [54]	2013	USA	Army	Sergeants Major Academy	R	149	No
Cosman [64]	2013	USA	Army	Military Academy	P	755 M, 136 F	No
Cowan [74]	2012	USA	Army	Trainees	P	1568 F	Yes (F)
Davey [76]	2015	UK	Marines		P	1090 M	No (M)
Davey [127]	2016	UK	Marines		P	1082 M	No (M)
Fallowfield [77]	2018	UK	Air Force	Recruits	P	990 M, 203 F	Yes
George [114]	2012	USA	Army	Combat medics	P	1230	No
Grier [78]	2017	USA	Army	Infantry brigades	R	4236 M	Yes (M)
Grier [178]	2011	USA	Army	Ordinance school students	P	4255	Yes (M), no (F)
Hall [179]	2017	UK	Army	Recruits	R	3050 M	Yes (M)
Hauret [180]	2018	USA	Army	Recruits BCT	P	1181	Yes (endurance)
Heller [181]	2020	USA	Army	Recruits BCT	R	227 F	Yes (F)
Jones [34]	2017	USA	Army	Recruits BCT	R	143,398 M, 41,727 F	Yes
Keenan [172]	2017	USA	Multiple	Special Forces	P	726	Yes
Kelly [80]	2000	USA	Navy	Recruits BCT	R	86 F	No (F)
Knapik [81]	2010	USA	Air Force	Recruits BCT	P	1042 M, 375 F	Yes
Knapik [145]	2006	USA	Army	Recruits BCT	P	1174 M, 898 F	Yes
Knapik [83]	2013	USA	Army	Brigade combat team^#^	P	805	No
Knapik [182]	2003	USA	Army		R	1414 M, 1166 F	Yes
Knapik [84]	2007	USA	Army	Band	R	159 M, 46 F	No
Knapik [85]	2001	USA	Army	Recruits	P	182 M, 168 F	Yes
Knapik [86]	2008	USA	Army	Paratrooper training	R	1677	Yes
Knapik [87]	2008	USA	Army	Recruits BCT	P	2147 M, 920 F	Yes
Knapik [183]	2009	USA	Army	Recruits BCT	P	2689 M, 1263 F	Yes
Knapik [88]	2009	USA	Marines	Recruits BCT	P	840 M, 571 F	Yes
Kodesh [163]	2015	Israel	n/a	Combat Fitness Instructor Course	P	158 F	Yes (F) (running)
Krauss [168]	2017	USA	Army	Recruits BCT	R	1900 F	Yes
Kuikka [36]	2013	Finland	Army	Conscripts	R	128,584	No
Kupferer [164]	2014	USA	Air Force	Trainees	R	141	Yes
Lisman [118]	2013	USA	Marines	Officer candidate training	P	874	Yes (running)
Martin [184]	2018	USA	Army	Light infantry division	R	6865	Yes
Mattila [37]	2007	Finland	n/a	Conscripts	R	133,943 M, 2044 F	Yes (invers)
Mattila [38]	2007	Finland	Army	Conscripts	P	149,750 M, 2345 F	Yes
Moran [149]	2013	Israel	Army	Recruits	P	44	No
Müller-Schilling [185]	2019	Germany	Army	Recruits	P	774	Yes
Munnoch [91]	2007	UK	Marines		P	1115 M	No (M)
Nye [151]	2016	USA	Air Force	Recruits BCT	R	67,525	Yes
Psaila [94]	2017	Malta	n/a	Recruits BCT	P	114 M, 13 F	Yes
Rauh [186]	2006	USA	Marines		P	824 F	Yes (F)
Reynolds [96]	2009	USA	Army	Infantry	P	181	Yes
Reynolds [97]	2002	USA	Army	Construction engineers & Combat artillery soldiers	P	313	No
Reynolds [98]	2000	USA	Marines	Winter mountain training	P	356	No
Robinson [57]	2016	UK	Army	Recruits	P	1810	Yes (running)
Rosendal [187]	2003	Denmark	n/a	Conscripts BCT	P	330	Yes
Ruohola [100]	2006	Finland	n/a	Recruits	P	756 M	Yes (M)
Sanchez-Santos [65]	2017	UK	Marines	Recruits	P	1082 M	No (M)
Schneider [58]	2000	USA	Army	Airborne Div	R	1214	Yes
Scott [122]	2015	USA	Army	Reserve Officer Training	R	165 M, 30 F	No
Sefton [137]	2016	USA	Army	Recruits IET	P	1788 M	Yes (M)
Shaffer [109]	2006	USA	Marines	Recruits BCT	R	2962 F	Yes (F)
Sharma [102]	2019	UK	Army	Infantry recruits	P	562 M	Yes (M)
Sharma [103]	2011	UK	Army	Infantry recruits	P	468 M	Yes (M)
Sillanpää [51]	2008	Finland	n/a	Conscripts	R	128,508 M	No (M)
Sormaala [39]	2006	Finland	n/a	Recruits	R	118,149	No
Taanila [123]	2010	Finland	n/a	Conscripts	P	944 M	Yes (M)
Taanila [59]	2012	Finland	Army	Conscripts	P	982 M	Yes (M)
Trone [105]	2014	USA	Marine Corp Air Force Army	Recruits BCT	R	900 M, 597 F	Yes
Välimäki [188]	2005	Finland	Army	Conscripts	P	179	Yes
Waterman [165]	2010	USA	Military Academy		R	10,511 person years	Yes (invers)
Wilkinson [60]	2009	UK	Army	Infantry	P	660	No
Wyss [68]	2014	Switzerland	Army	Recruits BCT	P	1676	No
Wyss [189]	2012	Switzerland	Army		R	459	Yes
Zhao [108]	2016	China	Army	Recruits	P	1398 M	No (M)

*BCT* basis combat training; *IET* initial entry training; *n/a* not available; *R* retrospective study; *P* prospective study; *M* male; *F* female; *(M)* risk factor only for males; *(F)* risk factor only for females

*Risk factor for musculoskeletal injuries (MSkI); ^#^Deployment; ^##^Shoulder, knee, low back

There is weak scientific evidence for genetic factors as a non-modifiable risk factor.

#### Late menarche

Seven studies focused on late menarche as a risk factor for MSkIs (Table 8). All of the research was conducted within different branches of the US Armed Forces. The sizes of the study populations ranged from 136 to 3758 participants. Two studies identified late menarche as a risk factor for MSkIs, and 5 studies did not find a significant association.

There is no scientific evidence for late menarche as a non-modifiable risk factor for MSkIs.

#### Muscular strength (lower)

Eleven studies focused on muscular strength as a risk factor for MSkIs (Table 8), although it was measured in different ways depending on the study. Most of the research was conducted within the US Army (5 studies) or the military of Finland (4 studies). Additional studies were conducted within the militaries of Switzerland and the UK (1 study from each country). The sizes of the study populations ranged from 95 to 152,095 participants. Six studies identified low muscular strength as a risk factor for MSkIs, while 5 studies did not find a significant association. Notably, two studies with more than 100,000 participants found an inverse association between muscular strength and the risk for MSkIs, the other study found no association, but this study focused on traumatic patellar luxation.

There is moderate scientific evidence for lower muscular strength as a modifiable risk factor.

#### Physical fitness (low)

Seventy-four studies focused on physical fitness, based on results from physical fitness tests, as a risk factor for MSkIs (Table 8). Most of the research was conducted in different branches of the US Armed Forces (45 studies); 12 studies were conducted within the military of the UK, and 9 were conducted within the military of Finland. The remaining studies were conducted within the militaries of Israel and Switzerland (2 studies each) as well as China, Denmark, Germany, and Malta (1 study each). The size of the study population ranged from 44 to 238,772 participants. Fifty studies identified low physical fitness as a risk factor for MSkIs. Out of these 50 studies, 4 studies explored low physical endurance. Two studies found an association between low physical fitness and an increased risk for MSkI, but not for both sexes, and 20 studies did not find a significant association. In two studies, there was an inverse effect; high physical fitness was associated with an increased risk for MSkIs. A meta-analysis that included 27 publications found that the relative risk is 2.34 (95% CI 2.02—2.70) for injuries incurred during training, as well as for personnel who perform in the bottom quartile or quintile when compared to their peers in the top quartile or quintile of physical fitness [25].

There is strong scientific evidence for low physical fitness as a modifiable risk factor for MSkIs. Low physical fitness has an increased relative risk of 2.34 for MSkIs.

#### Secondary amenorrhea

Eight studies focused on having no menses in the last months (secondary amenorrhea) as a risk factor for MSkIs (Table 9). All of the research was conducted within different branches of the US Armed Forces. The sizes of the study populations ranged from 86 to 2962 participants. Three studies identified secondary amenorrhea as a risk factor for MSkIs, and 5 studies did not find a significant association.

**Table 9:** Summary of studies that focused on secondary amenorrhea, sex, plantar pressure assessment (of walking gait), range of tibial rotation, tibia length, and waist circumference as risk factors for MskIs

Study	Publication year	Country	Branches	Unit/training	Study type	*n*	Risk factor^*^
Secondary amenorrhea							
Canham-Chervak [52]	2006	USA	Army	Recruits	P	746 F	No
Kelly [80]	2000	USA	Navy	Recruits BCT	R	86 F	No
Knapik [81]	2010	USA	Air Force	Recruits BCT	P	375 F	No
Knapik [82]	2013	USA	Army	Army military police training	P	553 F	Yes
Knapik [87]	2008	USA	Army	Recruits BCT	P	920 F	No
Knapik [88]	2009	USA	Marines	Recruits BCT	P	571 F	No
Rauh [186]	2006	USA	Marines		P	824 F	Yes
Shaffer [109]	2006	USA	Marines	Recruits BCT	R	2962 F	Yes
Sex (female)							
Allsopp [159]	2003	UK	Navy	Recruits	R	1287 M, 354 F	Yes
Anderson [71]	2017	USA	Army	Light Infantry	R	4384 M, 363 F	No
Bell [27]	2000	USA	Army	Recruits	P	861	No
Billings [160]	2004	USA	Air Force	Recruits BCT	R	2006	Yes
Blacker [161]	2008	UK	Army	Recruits	R	11,937 M, 1480 F	Yes
Bulathsinhala [49]	2017	USA	Army	Active duty	R	1,299,332	Yes
Burne [170]	2004	Australia	Military Academy		P	122 M, 25 F	Yes
Canham-Chervak [141]	2000	USA	Army	Recruits BCT	P	655 M, 498 F	Yes
Craig [40]	2000	USA	Army	Airborne Division	R	242,949 aircraft exits	Yes
Darakjy [8]	2006	USA	Army	Active duty	P	4101 M, 413 F	Yes
Fallowfield [77]	2018	UK	Air Force	Recruits	P	990 M, 203 F	Yes
Finestone [167]	2008	Israel	Army	Light infantry training	P	36 M, 99 F	No
Finestone [190]	2014	Israel	Army	Cadets	P	78 M, 227 F	Yes
Gam [191]	2005	Israel	n/a	Recruits	P	375 M, 138 F	Yes
Garnock [113]	2018	Australia	Navy	Recruits	P	95 M, 39 F	Yes
Gemmell [192]	2002	UK	Army	Recruits	R	11,907 M, 1483 F	Yes
George [114]	2012	USA	Army	Combat medics	P	1230	Yes
Havenetidis [143]	2011	Greece	n/a	Recruits	P	253	Yes
Hill [115]	2013	USA	Army	Active duty	R	83,323	No
Itskoviz [32]	2011	Israel	Army	Recruits	R	n/a	Yes
Knapik [81]	2010	USA	Air Force	Recruits BCT	P	1042 M, 375 F	No
Knapik [82]	2013	USA	Army	Army military police training	P	1838 M, 553 F	Yes
Knapik [83]	2013	USA	Army	Brigade Combat Team^#^	P	805	Yes (to be male)
Knapik [84]	2007	USA	Army	Band	R	159 M, 46 F	No
Knapik [85]	2001	USA	Army	Recruits	P	182 M, 168 F	Yes
Knapik [46]	2018	USA	Army	Recruits BCT	R	475,745 M, 107,906 F	Yes
Kupferer [164]	2014	USA	Air Force	Trainees	R	141	Yes
Mattila [37]	2007	Finland	n/a	Conscripts	R	133,943 M, 2044 F	Yes
Mattila [38]	2007	Finland	Army	Conscripts	P	149,750 M, 2345 F	Yes
Montain [45]	2013	USA	Army	Recruits BCT	R	421,461 M, 90,141 F	Yes
Nye [151]	2016	USA	Air Force	Recruits BCT	R	67,525	Yes
Owens [128]	2009	USA	Army, Marines, Navy, Air Force	Active duty	R	19,730	Yes
Roy [133]	2012	USA	Army	Brigade Combat Team^#^	P	246 M, 17 F	Yes
Scott [122]	2015	USA	Army	Reserve Officer Training	R	165 M, 30 F	No
Snedecor [193]	2000	USA	Air Force	Recruits	R	8656 M, 5250 F	Yes
Sormaala [39]	2006	Finland	n/a	Recruits	R	118,149	No
Waterman [165]	2010	USA	Military Academy		R	10,511 person years	Yes
Waterman [31]	2016	USA	Multiple	Active Duty	R	5,580,875	Yes
Yates [176]	2004	UK	Navy	Recruits	P	84 M, 40 F	Yes
Plantar pressure assessment (of walking gait)							
Finestone [112]	2011	Israel	Army	Elite infantry soldier	P	77 M	No (M)
Mahieu [148]	2006	Belgium	n/a	Recruits Royal Military Academy	P	69 M	Yes (M)
Nunns [150]	2016	UK	Marines	Recruits	P	160 M	No (M)
Rice [120]	2017	UK	Marines	Recruits	P	147 M	Yes^$^ (M)
Sharma [103]	2011	UK	Army	Infantry recruits	P	468 M	Yes (M)
Range of tibial rotation during running (lower)							
Nunns [150]	2016	UK	Marines	Recruits	P	160 M	Yes (M)
Tibia length (shorter)							
Beck [140]	2000	USA	Marines		P	624 M, 693 F	Yes
Finestone [112]	2011	Israel	Army	Elite infantry soldier	P	77 M	Yes (M)
Goss [194]	2006	USA	Military Academy	Cadets	R	1100	No^##^
Moran [149]	2013	Israel	Army	Recruits	P	44	No
Zhao [108]	2016	China	Army	Recruits	P	1398 M	No (M)^###^
Waist circumference (higher)							
Kupferer [164]	2014	USA	Air Force	Trainees	R	141	No
Nye [151]	2016	USA	Air Force	Recruits BCT	R	67,525	No
Taanila [123]	2010	Finland	n/a	Conscripts	P	944 M	Yes (M)
Taanila [59]	2012	Finland	Army	Conscripts	P	982 M	No (M)
Taanila [104]	2015	Finland	Army	Conscripts	P	1411 M	Yes (M)

*BCT* basis combat training; *IET* initial entry training; *n/a* not available; *R* retrospective study; *P* prospective study; *M* male; *F* female; *(M)* risk factor only for males; *(F)* risk factor only for females

*Risk factor for musculoskeletal injuries (MSkI); ^#^Deployment; ^##^Limb length inequality; ^###^Leg length; ^$^Pressure on digital V

There is insufficient scientific evidence for secondary amenorrhea as a modifiable risk factor.

#### Sex (female)

Thirty-eight studies focused on sex as a risk factor for MSkIs (Table 9). Most of the research was conducted within different branches of the US Armed Forces (24 studies). Additional studies were conducted within the militaries of Israel and the UK (4 studies each), Finland (3 studies), Australia (2 studies), and Greece (1 study). The sizes of the study populations ranged from 124 to 5,580,875 participants. Twenty-nine studies identified being female as a risk factor for MSkIs (when compared to males), 8 studies did not find a significant association between sex and MSkIs, and 1 study found a significant increase in MSkIs for males when compared to females.

There is strong scientific evidence that being female is a non-modifiable risk factor for MSkIs.

#### Plantar pressure assessment (of walking gait)

Five studies focused on plantar pressure assessment (of walking gait) as a risk factor for MSkIs (Table 9). Most of the research was conducted within different branches of the UK military (3 studies). Additional studies were conducted within the militaries of Belgium and Israel (1 study from each country). The study populations ranged from 69 to 468 participants. All studies included males only. Two studies identified a particular foot pressure pattern while walking as a risk factor for MSkIs, and two studies did not find a significant association. In one study, this association was only found for a pressure pattern involving the little toe (digitus V).

There is insufficient scientific evidence for specific plantar pressure patterns during walking as a modifiable risk factor.

#### Range of tibial rotation during running (lower)

Only one study focused on the range of tibial rotation (calculated as the difference between peak internal and external rotation) during running as a risk factor for MSkIs (Table 9). This study was conducted within the UK Marines. In this prospective study with 160 male participants, a lower range of tibial rotation during running (the difference between peak internal and external lower leg segment rotation) was identified as a risk factor for MSkIs.

There is weak scientific evidence for a lower range of tibial rotation during running as a modifiable risk factor.

#### Tibia length (shorter)

Four studies focused on tibia length as a risk factor for MSkIs (Table 9). The research was conducted within the IDF (2 studies) and within the US Marines (1 study) and within the army of China (1 study). The sizes of the study populations ranged from 44 to 1398 participants. Two studies identified a shorter tibia length as a risk factor for MSkIs, and the two studies did not find a significant association. Hence, one of these studies reported leg length, not tibia length.

There is insufficient scientific evidence for shorter tibia length as a modifiable risk factor.

#### Waist circumference (higher)

Five studies focused on high circumference as a risk factor for MSkIs (Table 9). Three studies were conducted within the military of Finland, and two were carried out within the US Air Force. The size of the study populations ranged from 141 to 67,525 participants. Two studies from Finland identified high circumference as a risk factor for MSkIs, while the other 3 studies did not find a significant association. Especially, the retrospective study by Nye et al. [151], with 67,525 participants, found no association between high waist circumference and an increased risk for MSkIs.

There is insufficient scientific evidence for a high waist circumference as a modifiable risk factor.

### Social factors

#### Education (lower)

Thirteen studies focused on education as a risk factor for MSkIs (Table 10). Nearly half of the research was conducted within different branches of the US Armed Forces (6 studies); the others were conducted within the militaries of Finland (4 studies), the UK (2 studies), and Israel (1 study). The sizes of the study populations ranged from 205 to 4029 participants. Five of the 13 studies identified a lower level of education as a risk factor for MSkIs, and 8 studies did not find a significant association between lower education and MSkIs. The definitions of lower education are different among the studies examined.

**Table 10:** Summary of studies that focused on education, marital status, race/ethnicity, rank, season of the year, and UV index as risk factors for MskIs

Study	Publication year	Country	Branches	Unit/training	Study type	*n*	Risk factor^*^
Education (lower)							
Canham-Chervak [52]	2006	USA	Army	Recruits	P	1156 M, 746 F	Yes
Fallowfield [77]	2018	UK	Air Force	Recruits	P	990 M, 203 F	Yes
George [114]	2012	USA	Army	Combat medics	P	1230	No
Givon [61]	2000	Israel	n/a		P	2306 M	No (M)
Knapik [84]	2007	USA	Army	Band	R	159 M, 46 F	No
Knapik [87]	2008	USA	Army	Recruits BCT	P	2147 M, 920 F	No
Knapik [88]	2009	USA	Marines	Recruits BCT	P	840 M, 571 F	No
Munnoch [91]	2007	UK	Marines		P	1115 M	No (M)
Pihlajamäki [93]	2019	Finland	n/a	Full duty	R	4029 M	No (M)
Reynolds [98]	2000	USA	Marines	Winter mountain training	P	356	Yes
Taanila [123]	2010	Finland	n/a	Conscripts	P	944 M	Yes (M)
Taanila [59]	2012	Finland	Army	Conscripts	P	982 M	Yes (M)
Taanila [104]	2015	Finland	Army	Conscripts	P	1411 M	No (M)
Marital status							
Canham-Chervak [52]	2006	USA	Army	Recruits	P	1156 M, 746 F	No
Hill [115]	2013	USA	Army	Active duty	R	83,323	Yes
Knapik [84]	2007	USA	Army	Band	R	159 M, 46 F	No
Knapik [87]	2008	USA	Army	Recruits BCT	P	2147 M, 920 F	Yes^#^
Knapik [88]	2009	USA	Marines	Recruits BCT	P	840 M, 571 F	No
Schneider [58]	2000	USA	Army	Airborne Div	R	1214	No
Race/ethnicity							
Bedno [72]	2013	USA	Army	IET	P	8456 M	No (M)^1^
Billings [160]	2004	USA	Air Force	Recruits BCT	R	2006	Yes^2^
Blacker [161]	2008	UK	Army	Recruits	R	11,937 M, 1480 F	Yes^3^
Bulathsinhala [49]	2017	USA	Army	Active duty	R	1,299,332	Yes^4^
Canham-Chervak [52]	2006	USA	Army	Recruits	P	1156 M, 746 F	No^5^
Cowan [74]	2012	USA	Army	Trainees	P	1568 F	No (F)^1^
Cowan [75]	2011	USA	Army	Recruits	P	7323	No^1^
Givon [61]	2000	Israel	n/a		P	2306 M	No (M)^6^
Grier [79]	2010	USA	Multiple		R	24,177 M	Yes^7^
Hughes [50]	2019	USA	Army	Active duty	R	120,730	Yes^8^
Kelly [80]	2000	USA	Navy	Recruits BCT	R	86 F	Yes^9^
Knapik [47]	2012	USA	Army	Recruits BCT	R	475,745 M, 107,906 F	Yes^10^
Knapik [146]	2007	USA	Army	Mechanics	R	518 M, 43 F	No^11^
Knapik [46]	2018	USA	Army	Recruits BCT	R	475,745 M, 107,906 F	Yes^10^
Knapik [87]	2008	USA	Army	Recruits BCT	P	2147 M, 920 F	No^12^
Knapik [88]	2009	USA	Marines	Recruits BCT	P	840 M, 571 F	No^13^
Lappe [55]	2005	USA	Army	Recruits BCT	R	4139 F	Yes^14^
Lappe [56]	2001	USA	Army	Recruits BCT	P	3758 F	Yes^15^
Lauder [90]	2000	USA	Army	Active duty	P	230 F	No (F)^16^
Montain [45]	2013	USA	Army	Recruits BCT	R	421,461 M, 90,141 F	Yes^17^
Owens [152]	2007	USA	n/a	Active duty	R	4451	Yes^18^
Owens [128]	2009	USA	Army, Marines, Navy, Air Force	Active duty	R	19,730	Yes^19^
Reynolds [96]	2009	USA	Army	Infantry	P	181	Yes^20^
Reynolds [97]	2002	USA	Army	Construction engineers & Combat artillery soldiers	P	313	Yes^21^
Reynolds [98]	2000	USA	Marines	Winter mountain training	P	356	Yes^22^
Waterman [31]	2016	USA	Multiple	Active Duty	R	5,580,875	Yes^19^
Wilkinson [60]	2009	UK	Army	Infantry	P	660	No^23^
Rank (lower)							
Canham-Chervak [52]	2006	USA	Army	Recruits	P	1156 M, 746 F	No
Craig [40]	2000	USA	Army	Airborne Division	R	242,949 aircraft exits	Yes
Darakjy [8]	2006	USA	Army	Active duty	P	4101 M, 413 F	Yes
Grier [79]	2010	USA	Multiple		R	24,177 M	No (M)
Hill [115]	2013	USA	Army	Active duty	R	83,323	Yes
Lauder [90]	2000	USA	Army	Active duty	P	230 F	No (F)
Owens [128]	2009	USA	Army, Marines, Navy, Air Force	Active duty	R	19,730	Yes
Reynolds [98]	2000	USA	Marines	Winter mountain training	P	356	Yes
Roy [133]	2012	USA	Army	Brigade Combat Team^##^	P	246 M, 17 F	No
Skeehan [139]	2009	USA	Army, Marine, Navy	Active duty^##^	R	3367	Yes
Wilkinson [60]	2009	UK	Army	Infantry	P	660	No
Season of the year (summer time)							
Jones [33]	2008	USA	Army	Ordinance school students	P	n/a	Yes^$^
Knapik [195]	2002	USA	Army	Recruits BCT	R	1543 M, 1025 F	Yes^$^
Mattila [196]	2006	Finland	n/a		P	213,500 person years	Yes^$$^
Taanila [197]	2009	Finland	Army	Conscripts	P	955 M	Yes (M)^$^
UV index (higher)							
Montain [45]	2013	USA	Army	Recruits BCT	R	421,461 M, 90,141 F	Yes

*BCT* basis combat training; *IET* initial entry training; *n/a* not available; *R* retrospective study; *P* prospective study; *M* male; *F* female; *(M)* risk factor only for males; *(F)* risk factor only for females

*Risk factor for musculoskeletal injuries (MSkI); ^#^Divorced or widowed; ^##^Deployment; ^$^Summer; ^$$^Summer and autumn

^1^White vs. Black vs. other; ^2^Other > African American > Hispanic > Caucasian; ^3^Caucasian > others; ^4^Non-Hispanic white > Hispanic > American Indian/Native Alaskan > Asian > Native Hawaiian/Pacific Islander > Non-Hispanic Black > others; ^5^White vs. Black vs. Hispanic; ^6^Ashkenazi vs. non-Ashkenazi; ^7^Black > (Native, Causasian, Asian, Hispanic, other); ^8^White > Black (and Asian, American Indian, other); ^9^Hispanic and Asian and other > white and African American; ^10^White, Hispanic, Asian, American Indian, other > Black; ^11^Caucasian vs. African American vs. other; ^12^White, Hispanic, Asian, American Indian, Black and others; ^13^White, Hispanic, Black, Other; ^14^Hispanic and White > Black, American Indians, Asian; ^15^All others races and White > Black; ^16^Hispanic and Asian > African-American or Caucasian; ^17^White, Hispanic, Asian, American Indian, others > Black; ^18^Black vs. White and others; ^19^White > others > Black; ^20^Caucasian > African-American, Hispanic, others; ^21^Caucasian was identified as a risk factor; ^22^White was identified as a risk factor; ^23^White vs. others

There is weak scientific evidence for a lower level of education as a non-modifiable risk factor for MSkIs.

#### Marital status

Six studies focused on marital status as a risk factor for MSkIs (Table 10). All of the research was conducted within different branches of the US Armed Forces (mostly in the army). The sizes of the study populations ranged from 205 to 83,323 participants. Only one study (with the largest number of participants examined) identified being married as a risk factor for MSkI. Another study identified being divorced or widowed as a risk factor for MSkIs. The remaining 4 studies did not find a significant association between marital status and MSkIs.

There is insufficient scientific evidence for marital status as a non-modifiable risk factor.

#### Race/ethnicity

Twenty-seven studies focused on race/ethnicity as a risk factor for MSkIs (Table 10). Most of the research was conducted within different branches of the US Armed Forces (24 studies); 2 studies were conducted within the militaries of the UK, and 1 was conducted in Israel. The sizes of the study populations ranged from 86 to 5,580,875 participants. Seventeen studies identified race/ethnicity as a risk factor for MSkIs, while 10 studies did not find a significant association. When only studies with more than 10,000 participants were taken into account (9 studies, total: 8,640,581 participants), all studies found an association between race/ethnicity and the risk for MSkIs, but the findings were contradictory in that there was no clear association as to which race/ethnicity was at the highest risk.

There is strong scientific evidence for race/ethnicity as a non-modifiable risk factor for MSkIs.

#### Rank (lower)

Eleven studies focused on rank as a risk factor for MSkIs (Table 10). All except one of the studies were conducted within different branches of the US Armed Forces, and the exception was conducted within the British Army. The sizes of the study populations ranged from 230 to 242,949 participants or aircraft exits. Six studies identified as having a lower rank as a risk factor for MSkIs, and 5 studies did not find a significant association between rank and MSkIs (3 of the 5 had less than 1000 participants).

There is weak scientific evidence for lower rank as a non-modifiable risk factor.

#### Seasons of the year (summertime)

Four studies focused on the seasons of the year as a risk factor for MSkIs (Table 10). Two studies were conducted within the Finnish armed forces and two within the US Army. The study populations ranged from 955 to 2568 participants, and one study examined 213,500 person-years. All 4 studies identified the effect of the season of the year as a risk factor for MSkIs, with a higher risk in the summer months.

There is strong scientific evidence for the season of the year (summertime) as a non-modifiable risk factor for MSkIs.

#### UV index (higher)

Only one study focused on the UV index (a surrogate for vitamin D exposure) as a risk factor for MSkIs (Table 10). This study was conducted within the US Army. In this retrospective study, with 511,602 participants, a higher UV index at a recruit’s home before basic combat training (BCT) was identified as a risk factor for MSkIs during BCT. The relative risk reduction for a lower UV index was small (0.92 and 0.89 vs. 1.00, *P* < 0.01).

There is weak scientific evidence for a higher UV index as a non-modifiable risk factor.

### Training factors

#### Equipment: running shoes

Only one study focused on running shoes as a risk factor for MSkIs (Table 11). This study was conducted within the US Armed Forces. In this prospective study, with 827 participants, no association between the kinds of running shoes and an increased risk for MSkIs could be identified.

**Table 11:** Summary of studies that focused on running shoes, participation in sports before military service, time available and participation rate in physical training, personal non-military training, unit training, training program content, and site as risk factors for MSkI

Study	Publication year	Country	Branches	Unit/training	Study type	*n*	Risk factor^*^
Running shoes							
Helton [198]	2019	USA	Military Academy	Cadets	P	827	No
Participation in sport before military service (no or low)							
Canham-Chervak [52]	2006	USA	Army	Recruits	P	1156 M, 746 f	Yes
Dash [66]	2012	India	Army	Recruits	P	8570	Yes
Finestone [112]	2011	Israel	Army	Elite Infantry soldier	P	77 M	Yes (only for ball sports)
Garnock [113]	2018	Australia	Navy	Recruits	P	95 M, 39 F	No (running)
Kelly [80]	2000	USA	Navy	Recruits BCT	R	86 F	No (F)
Knapik [82]	2013	USA	Army	Army military police training	P	1838 M, 553 F	Yes
Knapik [116]	2013	USA	Army	Combat engineer enlisted trainees	P	1633	Yes
Knapik [85]	2001	USA	Army	Recruits	P	182 M, 168 F	No
Knapik [87]	2008	USA	Army	Recruits BCT	P	2147 M, 920 F	Yes (M), no (F)
Knapik [88]	2009	USA	Marines	Recruits BCT	P	840 M, 571 F	Yes (M), no (F)
Lappe [55]	2005	USA	Army	Recruits BCT	R	4139 F	Yes (F)
Lappe [56]	2001	USA	Army	Recruits BCT	P	3758 F	Yes (F)
Lisman [118]	2013	USA	Marines	Officer candidate training	P	874	Yes
Monnier [119]	2019	Sweden	Marines	Training course	P	48 M, 5 F	No
Pihlajamäki [93]	2019	Finland	n/a	Full duty	R	4029 M	Yes (M)
Rauh [186]	2006	USA	Marines		P	824 F	Yes (F)
Rosendal [187]	2003	Denmark	n/a	Conscripts BCT	P	330	Yes
Sanchez-Santos [65]	2017	UK	Marines	Recruits	P	1082 M	Yes (M) (invers)
Scheinowitz [101]	2017	Israel	Army	Recruits	P	350 F	No (F)
Scott [122]	2015	USA	Army	Reserve Officer Training	R	165 M, 30 F	No
Taanila [104]	2015	Finland	Army	Conscripts	P	1411 M	Yes (M)
Trone [105]	2014	USA	Marine Corp Air Force Army	Recruits BCT	R	900 M, 597 F	Yes
Wang [106]	2003	China	n/a	Military Police Forces Training	R	805 M	Yes (M)
Zhao [108]	2016	China	Army	Recruits	P	1398 M	Yes (M)
Time available for taking part in physical training (low)							
Knapik [86]	2008	USA	Army	Paratrooper training	R	1677	No
Wyss [68]	2014	Switzerland	Army	Recruits BCT	P	1676	Yes
Participation rate in physical training (low)							
Knapik [84]	2007	USA	Army	Band	R	159 M, 46 F	Yes
Martin [184]	2018	USA	Army	Light Infantry division	R	6865	Yes
Roy [133]	2012	USA	Army	Brigade Combat Team^#^	P	246 M, 17 F	No
Roy [121]	2014	USA	Army	Active duty	R	625 F	Yes (F)
Scott [122]	2015	USA	Army	Reserve Officer Training	R	165 M, 30 F	No
Wilkinson [60]	2009	UK	Army	Infantry	P	660	No
Personal non-military training (high amounts)							
George [114]	2012	USA	Army	Combat medics	P	1230	No
Grier [78]	2017	USA	Army	Infantry brigade	R	4236 M	Yes (M) (invers)
Lisman [118]	2013	USA	Marines	Officer candidate training	P	874	No
Moran [62]	2012	Israel	Army	Recruits of elite combat unit	P	116	Yes
Rappole [132]	2018	USA	Army	Active duty	R	368 F	Yes (F) (invers)
Shaffer [109]	2006	USA	Marines	Recruits BCT	R	2962 F	Yes (F)
Taanila [59]	2012	Finland	Army	Conscripts	P	982 M	No (M)
Wyss [68]	2014	Switzerland	Army	Recruits BCT	P	1676	Yes
Unit training (high amounts)							
Grier [78]	2017	USA	Army	Infatery brigades	R	4236 M	Yes (M)
Knapik [199]	2011	USA	Army	Recruits BCT	P	2072	Yes
Lauder [90]	2000	USA	Army	Active duty	P	230 (F)	Yes (F)
Lisman [118]	2013	USA	Marines	Officer candidate training	P	874	No
Moran [149]	2013	Israel	Army	Recruits	P	44	Yes
Nye [151]	2016	USA	Air Force	Recruits BCT	R	67,525	Yes
Roos [99]	2015	Switzerland	Army	Recruits	P	651 M	Yes (M)
Roy [177]	2012	USA	Army	Brigade Combat Team	R	593	No
Schuh [200]	2017	USA	Army	Infantry soldiers	R	831	Yes
Scott [122]	2015	USA	Army	Reserve Officer Training	R	165 M, 30 F	Yes
Wang [106]	2003	China	n/a	Military Police Forces Training	R	805 M	No (M)
Training program content							
Knapik [201]	2005	USA	Army	Recruits BCT	P	1142 M, 825 F	Yes
Kovcan [67]	2019	Slovenia	Army	Infantry, active duty	R	118 M, 11 F	No
Rappole [132]	2018	USA	Army	Active duty	R	368 F	Yes^1^
Waterman [165]	2010	USA	Military Academy Students		R	10,511 person years	Yes
Training site							
Blacker [161]	2008	UK	Army	Recruits	R	11,937 M, 1480 F	Yes
Givon [61]	2000	Israel	n/a		P	2306 M	No (M)
Grier [79]	2010	USA	Multiple		R	24,177 M	Yes (M)
Jones [33]	2008	USA	Army	Ordinance school students	P	n/a	Yes
Schneider [58]	2000	USA	Army	Airborne Div	R	1214	No
Wilkinson [60]	2009	UK	Army	Infantry	P	660	No

*BCT* basis combat training; *n/a* not available; *R* retrospective study; *P* prospective study; *M* male; *F* female; *(M)* risk factor only for males; *(F)* risk factor only for females

^#^Deployment; ^1^Unit resistance training was associated with higher risk of MSkI; *Risk factor for musculoskeletal injuries (MSkI)

There is no scientific evidence for the kinds of running shoes as a modifiable risk factor.

#### Participation in sports before military service (no or low)

Twenty-four studies focused on a history of participation in sports before military service as a risk factor for MSkIs (Table 11). Most of the research was conducted among recruits or those new to military service within different branches of the US Armed Forces (13 studies). The militaries of China, Finland, and Israel conducted 2 studies each; the remaining studies were conducted within the militaries of Australia, Denmark, India, Sweden, and the UK (1 study each). The sizes of the study populations ranged from 53 to 8570 participants. Fifteen studies identified no or low participation in sports before military service time as a risk factor for MSkIs, and 6 studies (all with fewer than 350 participants) did not find a significant association. In two studies, an association was found only for men, and in another study, an inverse association was found (higher participation in a sport before military service was a risk factor for MSkIs).

There is strong scientific evidence for no or low participation in sports before military service time as a non-modifiable risk factor for MSkIs.

#### Physical training: available participation time (low)

Two studies focused on the amount of time available to take part in physical training as a risk factor for MSkIs (Table 11). The research was conducted within the US Army (1 study) and the army of Switzerland (1 study). The sizes of the study populations were 1677 and 1676 participants. The study from Switzerland found an association between having little time for physical training and an increased risk for MSkIs, while the study from the US military did not show a significant association.

There is insufficient scientific evidence for having little time available for taking part in physical training as a modifiable risk factor.

#### Physical training: participation rate (low)

Six studies focused on participation in physical training as a risk factor for MSkIs (Table 11). Most of the research was conducted within different branches of the US Armed Forces (5 studies). An additional study was conducted within the military of the UK. The study populations ranged from 195 to 6865 participants. Three studies identified a low participation rate in physical training as a risk factor for MSkIs, and 3 studies did not find a significant association.

There is insufficient scientific evidence for the participation rate in physical training as a modifiable risk factor.

#### Physical training: personnel, non-military training (high amounts)

Eight studies focused on high amounts of training during free time (non-military training) as a risk factor for MSkIs (Table 11). Most of the research was conducted within the army and the Marines Corp of the US Armed Forces (5 studies in total). Additional studies were conducted within the militaries of Finland, Israel, and Switzerland (1 study from each country). The sizes of the study populations ranged from 116 to 4236 participants. Three studies identified a high amount of personal training during free time as a risk factor for MSkIs, and 3 studies did not find a significant association. Two studies found an inverse effect; a low amount of personal training was associated with an increased risk of MSkIs.

There is insufficient scientific evidence for high amounts of personnel training during free time as a modifiable risk factor.

#### Physical training: unit training (high amount)

Eleven studies focused on physical training during unit training as a risk factor for MSkIs (Table 11). Most of the research was conducted within different branches of the US Armed Forces (8 studies). Additional studies were conducted within the militaries of China, Israel, and Switzerland (1 study from each). The study populations ranged from 44 to 67,525 participants. Eight studies identified a high amount of training during unit training as a risk factor for MSkIs, whereas 3 studies did not find a significant association.

There is strong scientific evidence for high amounts of training during unit training as a modifiable risk factor for MSkIs.

#### Training program content

Four studies focused on different training program content as a risk factor for MSkIs (Table 11). Three studies were conducted within the US Armed Forces and 1 in the Army of Slovenia. The sizes of the study populations ranged from 129 to 1967 participants. One study included a total of 10,511 person-years. Three studies identified that different training program content could be a risk factor for MSkIs, and the smallest study found no association.

There is weak scientific evidence for training program content as a modifiable risk factor.

#### Training site

Six studies focused on the training site as a risk factor for MSkIs (Table 11). The studies were conducted within the militaries of the US Armed Forces (3 studies), the UK (2 studies), and Israel (1 study). The sizes of the study populations ranged from 660 to 24,177 participants. Three studies identified the training site as a risk factor for MSkIs (two of these studies had more than 10,000 participants), and 3 studies did not find a significant association between the training site and MSkIs. It should be taken into account that the training site is a combination of many different factors (e.g., training situation, climate, infrastructure, etc.), so it is very difficult to identify the true factor that influenced the MSkI risk.

There is weak scientific evidence for training sites as a possibly modifiable risk factor.

### Risk factor classification

In sum, 57 potential risk factors for MSkIs in the military were identified. Twenty-one factors were classified as risk factors with a strong or moderate association with an increased risk for MSkIs. For 14 other potential risk factors, an association was possible, but the evidence in the scientific literature was considered weak. For the final 22 potential risk factors, the evaluation showed either insufficient evidence or no evidence. As such, they cannot be classified as risk factors for an increased risk for MSkIs at this time (Table 12).

**Table 12:** Summary of all factors and categorization in five scientific evidence grades (sorted alphabetically)

Strong	Moderate	Weak	Insufficient	No
Body fat (higher) (m)	Age (nm)	Balance (low) (m)	Alcohol intake (m)	Ankle dorsiflexion (limited) (nm)
Branch (nm)	Foot type (nm)	Current illness (nm)	Available participation time (low) (m)	Body height (higher) (nm)
Load carriage (m)	Length of service (nm)	Genetic factors (nm)	BMI in general (m)	Equipment: running shoes (m)
Military occupational specialty (nm)	Muscular strength (lower) (m)	Prescription of non-steroidal anti-inflammatory drugs (m)	Body weight (higher) (m)	Late menarche (nm)
Obesity (m)	Previous deployment (nm)	Prior pregnancy (nm)	Bone (mineral) density (low) (nm)	Prescription of contraceptive (m)
Overweight (m)	Vitamin D status (low) (m)	Range of tibial rotation during running (lower) (m)	Calcium intake (low) (m)	Status (active vs. reserve) (nm)
Participation in sports before military service (no or low) (nm)		Rank (lower) (nm)	Education (lower) (nm)	Vegetables consumption (m)
Physical fitness (low) (m)		Serum iron/serum ferritin (lower) (m)	External rotation of hip (higher) (nm)	
Previous MSkI (nm)		Sleep time (reduced) (m)	Flexibility (lower) (m)	
Race/ethnicity (nm)		Training program content (m)	Marital status (nm)	
Season of the year (summer time) (nm)		Training site (m)	Milk consumption (low) (m)	
Sex (female) (nm)		UV index (higher) (nm)	Participation rate in physical training (m)	
Smoking (m)		Vegetarian diet (m)	Personal non-military training (high amounts) (m)	
Underweight (m)		Waist circumference (higher) (m)	Plantar pressure assessment (of walking gait)	
Unit training (high amount) (m)			Secondary amenorrhoe (m)	
			Tibial length (shorter) (m)	

*m* modifiable; *nm* non-modifiable

**Fig. 2:**
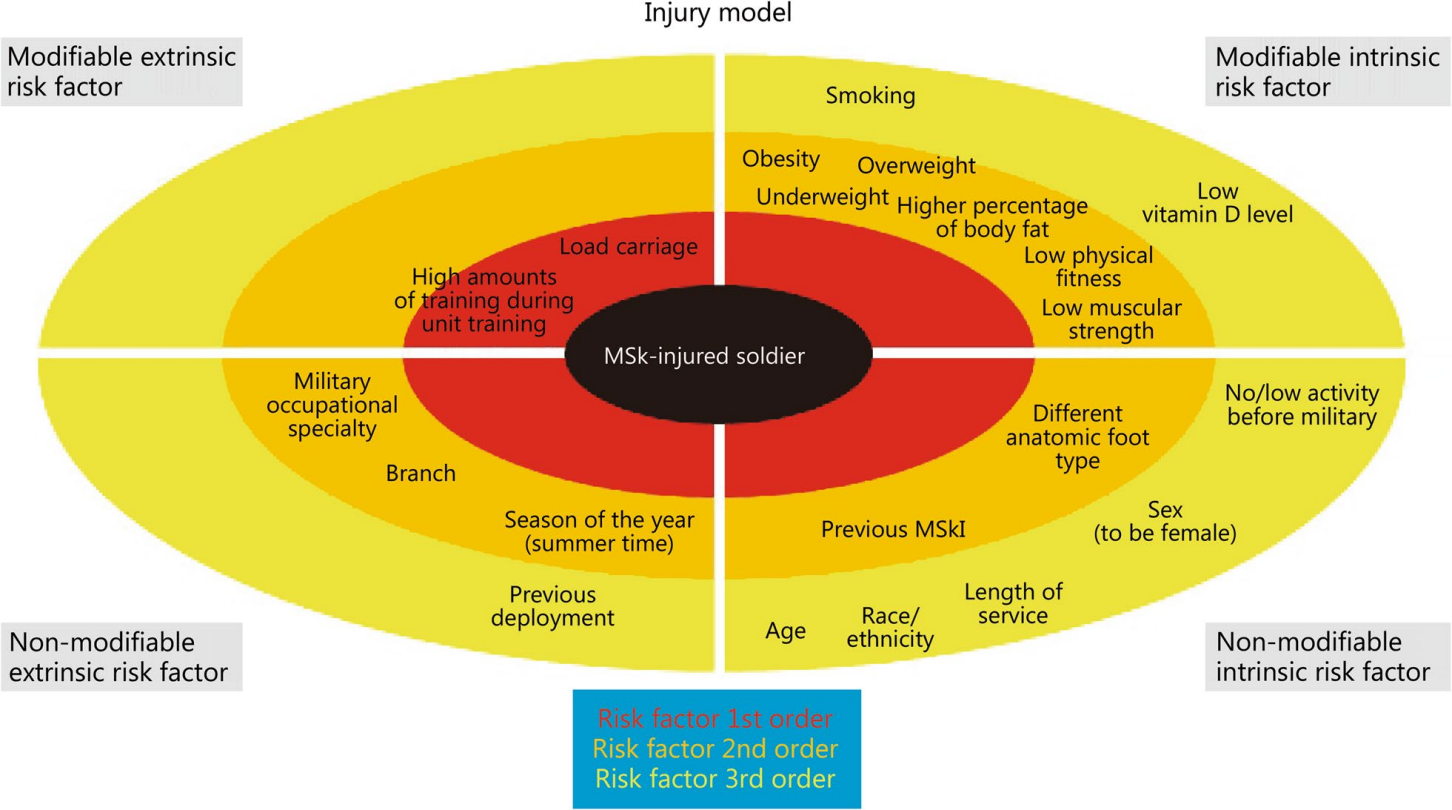
Injury model with a classification in 1st, 2nd and 3rd order

Based on this systematic literature review and an in-depth analysis, the NATO HFM-283 Research Task Group developed a model to classify the different risk factors identified. The classification model was based upon the rationale that some risk factors directly increase MSkI risk, whereas others merely increase the risk for MSkIs indirectly as a cofactor. As an example of a direct factor (1st order), high amounts of training during unit training increase the total volume of load placed upon the biological tissues of the soldier, directly resulting in injury. Alternatively, as an example of a cofactor, low vitamin D levels may lead to lower bone density, which may result in lower tissue resilience, which in turn may cause an MSkI due to the training load now exceeding the soldier’s reduced tissue capacity. The term “order” was used to classify how close each risk factor was to a direct cause of injury. A 1st-order risk factor was thought to be most closely related to injury, whereas a 3rd-order factor was thought to follow a path through multiple cofactors. Table 12 shows all risk factors categorized as 1st, 2nd, or 3rd order of importance. Additionally, the model includes the established concepts of modifiable/non-modifiable and extrinsic/intrinsic risk factors. This prioritizing classification model may guide the planning and implementation of intervention strategies, introducing the notion that a larger risk reduction can likely be achieved if risk factors in a higher order are targeted (Fig. 2).

## Discussion

This review is the qualitative systematic review of studies on risk factors for MSkIs in the military that has attempted to be all-inclusive. With a total of 179 original papers and 3 meta-analyses from the past two decades, a very large number of studies on MSkIs in the military were included. A total of 57 different risk factors were identified and evaluated.

The approach used in this study identified more risk factors for MSkIs in the military than previously reported [15–26]. The aim was to have an overview of all risk factors in one place. Further, the project is one of the first to include the classification of risk factors for MSkIs in the military into modifiable or non-modifiable categories. This additional distinction (modifiable vs. non-modifiable) helps us to understand which risk factors can be addressed and which ones cannot be addressed when an intervention is planned.

In addition to listing all potential risk factors, the members of the multidisciplinary expert panel assessed the combined evidence presented for each risk factor on a five-grade scale (strong evidence to no evidence). The number of participants (e.g., > 10,000 subjects) significantly influenced the evaluation of available evidence. Some classifications of available evidence had to be made based on a small number of studies with a small number of participants. The final rating also included the subjective professional experience (opinion) of the experts on the panel.

This review introduces a new injury model for the military, incorporating the established principles of modifiable vs. non-modifiable and intrinsic vs. extrinsic risk factors. The model clearly illustrates differences between risk factors; some increase the risk for MSkIs directly (1st order), whereas others influence the injury risk only indirectly (2nd or 3rd order). The model may explain why many of the interventions that have been attempted over the past decades to reduce MSkIs were not successful. In fact, a systematic review of successful interventions in reducing MSkIs in the military [6] shows that the only successful interventions are those that target 1st- and 2nd-order modifiable risk factors (i.e., in the upper half of the model).

Hence, most of the scientific publications are from the US Armed Forces, with studies conducted by other countries much less frequently. As such, the findings may not be generalizable across all nations. In addition, most studies focused on one branch of the armed forces—the army—which might not be representative of all service branches. Transferring the information from one country to another or from one military branch to another must be done with great caution.

Even with the very broad systematic approach used in this review, no studies on psychological, cognitive, and/or behavioral risk factors for MSkIs in the military could be identified. In civilian sports, these risk factors have been reported for several years [202, 203]. It is possible that the search terms used in this review did not allow for psychological factors to be identified or the psychosocial aspects of injuries.

This review has several limitations. First, the method used is a variation of the strict PRISMA protocol for systematic reviews. The group of coauthors decided that the topic at hand deserved a broad approach, including all possible risk factors and all military studies, even those with a potentially poor scientific design. In addition, it was decided to include the multidisciplinary, professional experience of the group as a subjective element in assessing the level of evidence per risk factor reported. Second, all studies before 2000 were excluded. This was decided because training schedules and conditions in the militaries have changed significantly over the past two decades and anticipated that including studies from before 2000 would not yield additional, currently relevant insights. Third, this review did not include studies on risk factors for MSkIs in civilian sports activities. Although some of the risk factors for civilian sports injuries are the same, the military training environment has many unique aspects that make risk factors for MSkIs not comparable to civilian sports. Fourth, differences in how the risk factors were measured (e.g., self-report vs. direct measurements) or the potential interrelationships between risk factors (e.g., that the strong evidence for sex as a risk factor may be related to differences in the percentage of body fat or previous physical activity before service between the sexes) were not considered when assigning the level of evidence for each risk factor. However, these issues were taken into account when depicting the 1st-, 2nd-, or 3rd-order level of the risk factors in the model. Fifth, this review did not include calculated effect sizes or a meta-analysis of every risk factor. Of course, this could further enhance the scientific value of the current work. The authors propose that future scientific evaluations can now be done, concentrating on the risk factors that have been identified as high order and modifiable in this work.

## Conclusions

This systematic review presents an all-inclusive, graded overview of risk factors for MSkIs in the military. Experts with a multidisciplinary background, from a total of seven nations as part of the NATO Research Task Group, introduced a new prioritizing injury model for the military. The model provides a foundation for understanding which risk factors would be most important to address and in which order when an intervention is planned.

## Supplementary Information

**Additional file 1**. MESH search term.

## Data Availability

All data generated or analyzed in this review are included in the published article.

## Abbreviations

BCT: Basis combat training
DNBI: Disease and nonbattle injury
IET: Initial entry training
MSkIs: Musculoskeletal injuries
MOS: Military occupational specialties
NSAID: Nonsteroidal anti-inflammatory drugs
RTG: Research Task Group
STO: Science and Technology Organization
