# Supplementary material for: Risk factors for musculoskeletal injuries in the military: a qualitative systematic review of the literature from the past two decades and a new prioritizing injury model
Source: Mil Med Res. 2021 Dec 10;8:66. doi: 10.1186/s40779-021-00357-w (PMC8662851; doi:10.1186/s40779-021-00357-w)
Supplement: Supplementary file 1 — Additional file 1. MESH search term. [file 40779_2021_357_MOESM1_ESM.doc]

**Additional file 1** MESH search term

(((((("militaries"[All Fields] OR "military personnel"[MeSH Terms]) OR ("military"[All Fields] AND "personnel"[All Fields])) OR "military personnel"[All Fields]) OR "military"[All Fields]) OR "military s"[All Fields]) AND (((((((((((("injurie"[All Fields] OR "injuried"[All Fields]) OR "injuries"[MeSH Subheading]) OR "injuries"[All Fields]) OR "wounds and injuries"[MeSH Terms]) OR ("wounds"[All Fields] AND "injuries"[All Fields])) OR "wounds and injuries"[All Fields]) OR "injurious"[All Fields]) OR "injury s"[All Fields]) OR "injuryed"[All Fields]) OR "injurys"[All Fields]) OR "injury"[All Fields]) OR ((((((("injuries"[MeSH Subheading] OR "injuries"[All Fields]) OR "trauma"[All Fields]) OR "wounds and injuries"[MeSH Terms]) OR ("wounds"[All Fields] AND "injuries"[All Fields])) OR "wounds and injuries"[All Fields]) OR "trauma s"[All Fields]) OR "traumas"[All Fields]))) AND ((("basic"[All Fields] OR "basics"[All Fields]) AND ((((((((("education"[MeSH Subheading] OR "education"[All Fields]) OR "training"[All Fields]) OR "education"[MeSH Terms]) OR "train"[All Fields]) OR "train s"[All Fields]) OR "trained"[All Fields]) OR "training s"[All Fields]) OR "trainings"[All Fields]) OR "trains"[All Fields])) OR (((((("physical examination"[MeSH Terms] OR ("physical"[All Fields] AND "examination"[All Fields])) OR "physical examination"[All Fields]) OR "physical"[All Fields]) OR "physically"[All Fields]) OR "physicals"[All Fields]) AND ((((((((("education"[MeSH Subheading] OR "education"[All Fields]) OR "training"[All Fields]) OR "education"[MeSH Terms]) OR "train"[All Fields]) OR "train s"[All Fields]) OR "trained"[All Fields]) OR "training s"[All Fields]) OR "trainings"[All Fields]) OR "trains"[All Fields])))
